# Active microbial population dynamics and life strategies drive the enhanced carbon use efficiency in high-organic matter soils

**DOI:** 10.1128/mbio.00177-24

**Published:** 2024-02-20

**Authors:** Qicheng Xu, Ling Li, Junjie Guo, Hanyue Guo, Manqiang Liu, Shiwei Guo, Yakov Kuzyakov, Ning Ling, Qirong Shen

**Affiliations:** 1 Jiangsu Collaborative Innovation Center for Solid Organic Waste Resource Utilization, Nanjing Agricultural University, Nanjing, China; 2 Centre for Grassland Microbiome, State Key Laboratory of Grassland Agro-ecosystems, College of Pastoral Agricultural Science and Technology, Lanzhou University, Lanzhou, Gansu, China; 3 Department of Soil Science of Temperate Ecosystems, University of Gottingen, Göttingen, Germany; 4 Department of Agricultural Soil Science, University of Gottingen, Göttingen, Germany; 5 Peoples Friendship University of Russia (RUDN University), Moscow, Russia; University of California at Irvine, Irvine, CA, USA; Northern Arizona University, Flagstaff, AZ, USA

**Keywords:** carbon use efficiency, organic fertilization, qSIP, population dynamics, life strategy

## Abstract

Microbial carbon use efficiency (CUE) is a critical parameter that controls carbon storage in soil, but many uncertainties remain concerning adaptations of microbial communities to long-term fertilization that impact CUE. Based on H_2_^18^O quantitative stable isotope probing coupled with metagenomic sequencing, we disentangled the roles of active microbial population dynamics and life strategies for CUE in soils after a long-term (35 years) mineral or organic fertilization. We found that the soils rich in organic matter supported high microbial CUE, indicating a more efficient microbial biomass formation and a greater carbon sequestration potential. Organic fertilizers supported active microbial communities characterized by high diversity and a relative increase in net growth rate, as well as an anabolic-biased carbon cycling, which likely explains the observed enhanced CUE. Overall, these results highlight the role of population dynamics and life strategies in understanding and predicting microbial CUE and sequestration in soil.

**IMPORTANCE:**

Microbial CUE is a major determinant of global soil organic carbon storage. Understanding the microbial processes underlying CUE can help to maintain soil sustainable productivity and mitigate climate change. Our findings indicated that active microbial communities, adapted to long-term organic fertilization, exhibited a relative increase in net growth rate and a preference for anabolic carbon cycling when compared to those subjected to chemical fertilization. These shifts in population dynamics and life strategies led the active microbes to allocate more carbon to biomass production rather than cellular respiration. Consequently, the more fertile soils may harbor a greater microbially mediated carbon sequestration potential. This finding is of great importance for manipulating microorganisms to increase soil C sequestration.

## INTRODUCTION

Soil microbes utilize readily available organic compounds to multiply and ultimately contribute to the recalcitrant soil organic matter (SOM) pool fraction in the form of microbial necromass (1). Model simulations suggest that microbial necromass C may comprise up to 80% of the soil organic C (2). Thus, the proportion of C that a microbe commits toward growth relative to uptake (the sum of growth and respiration), also known as microbial carbon use efficiency (CUE) (3–5), is an important indicator of the potential of microbially mediated C sequestration in soils. A high CUE reflects efficient microbial biomass production and subsequently more microbial necromass, which benefits the stable SOM pool (4). Alternatively, elevated CUE improves microbial biomass production and subsequent extracellular enzyme production, which could ultimately lead to soil organic carbon (SOC) loss over time (1, 6). A recent study employing global-scale data sets, a microbial-process explicit model, data assimilation, deep learning, and meta-analysis revealed a positive correlation between CUE and SOC content (7). However, the mechanistic understanding of microbial processes underlying the increase in CUE with SOC remains poorly resolved.

In general, the soil is regarded as an oligotrophic environment and, as such, soil microorganisms are continuously exposed to C starvation and frequently to nutrient limitations (8, 9). Fertilization is an important soil management that profoundly impacts microbial life by directly supplying utilizable resources (10, 11). Compared with long-term mineral-only fertilized soils, organic fertilization provides both accessible nutrients and diverse organic C resources (10, 12, 13). Over the long term, soil amendments with only mineral fertilizers result in C limitation. Conversely, as one would expect, organically amended soils contain a higher level of SOM content (9). Under this circumstance, microbes can shift their ecological strategies to adapt to soil environments with distinct C storage patterns (e.g., variations in the quantity of SOM) due to phenotypic plasticity.

When microbial communities are acclimated to conditions associated with long-term organic fertilization, they are more diverse and have higher abundances of fast-growing species (14). Traditionally, microbes adapted to resource-abundant environments have been considered to have fast reproduction but low CUE (15, 16). Conversely, microbes that favor resource-limited environments most often have slow reproduction rates and high CUE (15, 16). This tends to imply a negative correlation between growth rate and CUE, aligning with the ruderal strategy, where fast growth rates are typically negatively related to high yields (17). However, emerging evidence has demonstrated that the trade-off between microbial CUE and growth rate is condition dependent (18, 19). For example, the width of a substance utilization spectrum may have an overwhelming effect on CUE rather than the growth rate (18). Species that specialize in a specific C resource have higher C use efficiencies than generalists that require more cellular machinery to utilize a larger array of substrates. A lab study involving 23 soil isolates showed that bacterial taxa with rapid growth were more efficient in C utilization than slow-growing forms (20). In addition, growth rate measurements are commonly conducted under laboratory conditions and, as such, may not reflect the actual growth rates of microbes in the field (17). In this study, (i) we estimated microbial population dynamics *in situ* within complex soil environments to examine the trade-off between microbial growth characteristics and CUE.

In addition to population dynamics, there is also a potential trade-off between resource acquisition and high CUE (17). When encountered with C limitation in soils under the application of mineral-only fertilizers, microbes manufacture larger amounts of extracellular enzymes in order to mineralize organic matter (21) or would develop the ability to use rare or complex organic molecules that are difficult to hydrolyze. This resource acquisition process results in less efficient C utilization with low CUE because polymerizing amino acid production (e.g., extracellular enzyme) requires substantial energy investment (21). In contrast, long-term organic fertilization might select soil microbes that have a more efficient C utilization due to reduced energy costs for C acquisition and stress tolerance. Based on these relationships, we hypothesize that (ii) microorganisms in organic C-rich soils have higher CUE due to reduced energy cost for resource acquisition.

**Fig 1:**
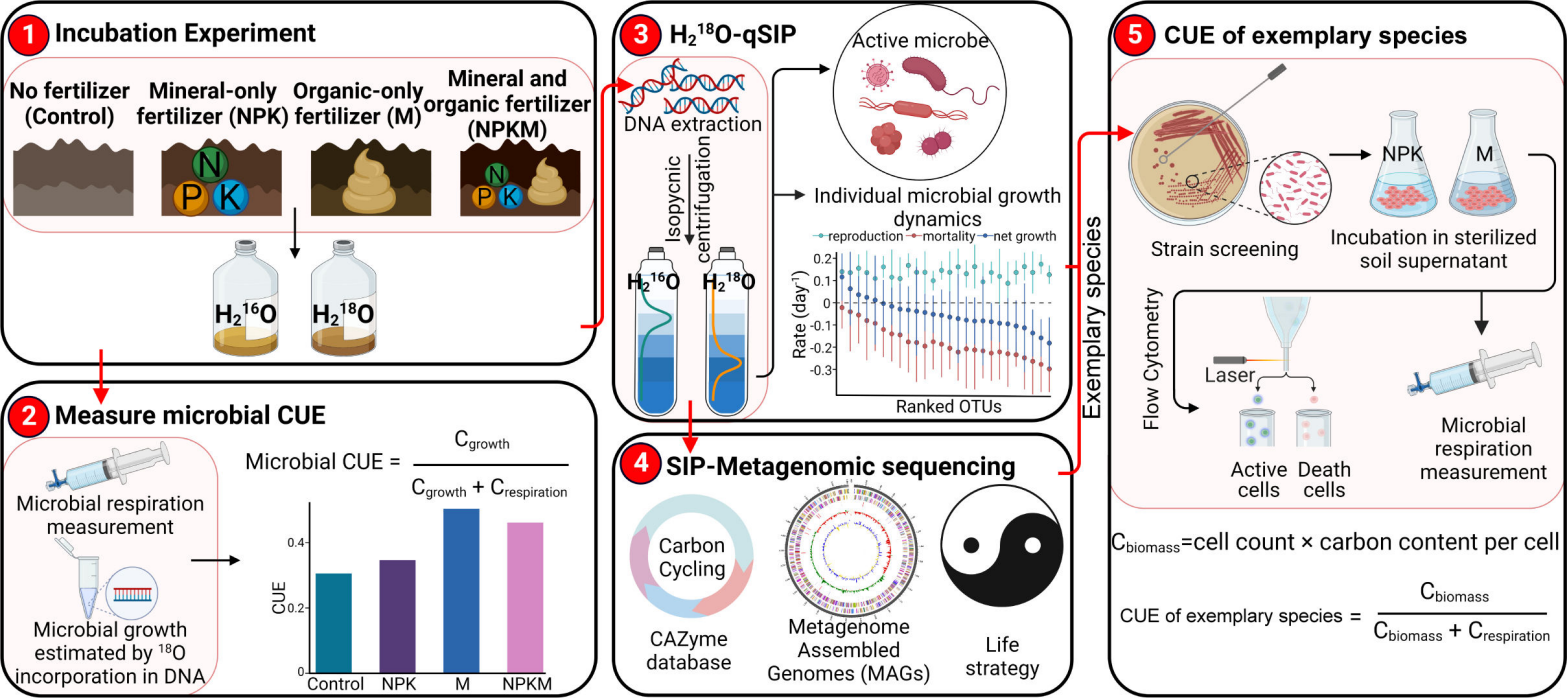
Overview of the experiment design and key analyses. The substrate-independent ^18^O tracing method was employed to determine microbial CUE in soils undergoing long-term fertilization regimes. Coupled with quantitative stable isotope probing (qSIP), we determined the active bacterial community and estimated the bacterial reproduction, mortality, and net growth rates Metagenomic sequencing was used to identify active microbial ecological processes at the functional level and to recover genomes of exemplary taxa (e.g., taxa with fast net growth rate) related to C cycling. The exemplary taxa were further isolated and incubated in different fertilization environments to test the effects of fertilization on microbial CUE. SIP, stable isotope probing.

Microbial CUE is often estimated by substrate-dependent methods, i.e., the incorporation and utilization of specific ^13^C- or ^14^C-labeled substances (3, 5). This approach defines microbial CUE within the constraint of a selected substrate, which cannot reflect the microbial use efficiency of C derived from the broad range of organic compounds present in the soil. Herein, the substrate-independent ^18^O tracing method was employed to determine microbial CUE in soils undergoing long-term fertilization regimes under mineral and/or organic fertilizer for more than 35 years (Fig. 1). This method tracks the utilization of multiple compounds within soil organic matter and estimates microbial CUE based on the incorporation of ^18^O-labeled water into DNA during microbial growth. This method is made possible because water oxygen is always required for polymerase reactions when cells are dividing (19, 22, 23). Coupled with quantitative stable isotope probing (qSIP), the active bacterial community was determined, and the bacterial reproduction, mortality, and net growth rates were estimated at both the taxon and community levels (Fig. 1). This approach helps pinpoint active microbial populations and species with distinct growth characteristics that contribute substantially to the energy and matter fluxes in soil. In addition, metagenomic sequencing was employed to identify active microbial ecological processes at the functional level and to recover genomes of exemplary taxa (e.g., taxa with fast net growth rate, Fig. 1). The exemplary taxa were further isolated and incubated in different fertilization environments to test the effects of fertilization on microbial CUE (Fig. 1). This provided a significantly deeper understanding regarding the ecological roles of active microbes in the context of CUE, population dynamics, and life strategies, and built a foundation for manipulating microorganisms in order to increase C-sequestration potential in soil.

## RESULTS

### Microbial biomass production and CUE

**Fig 2:**
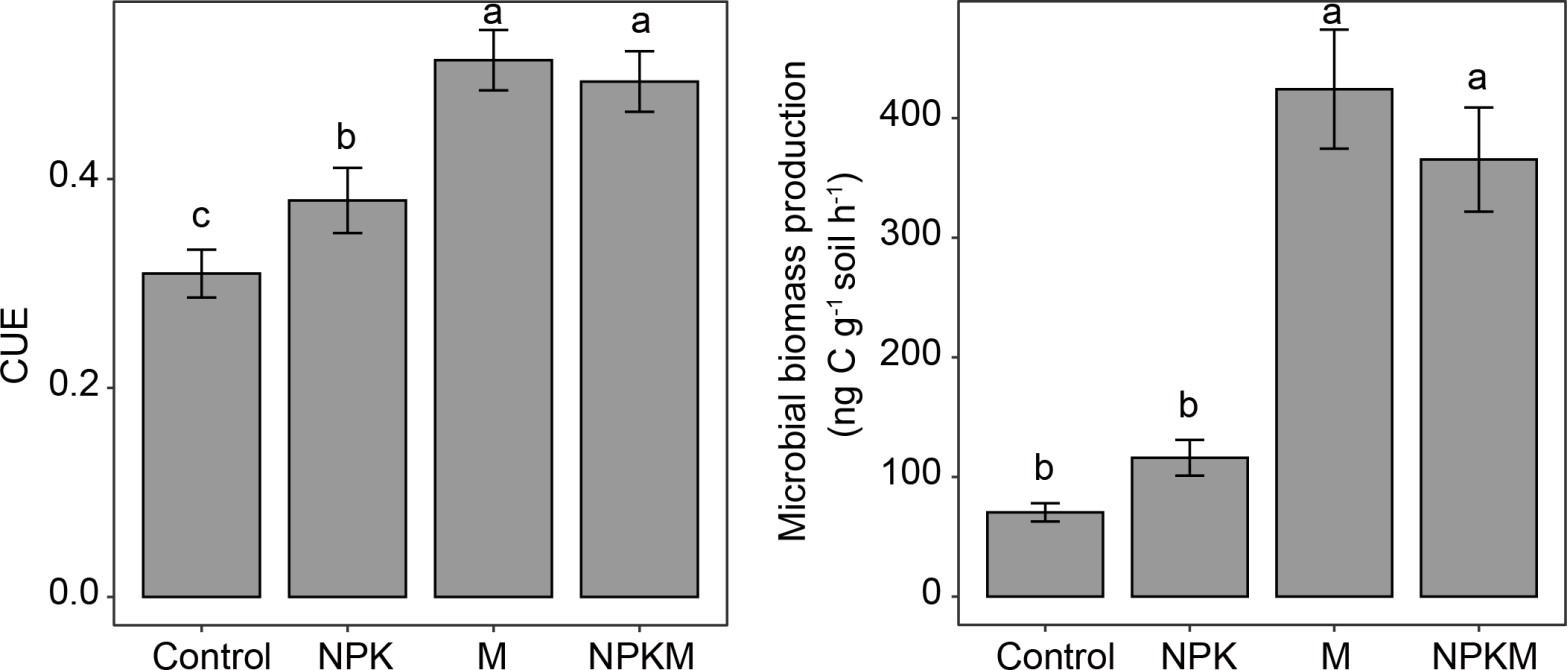
Microbial carbon use efficiency (CUE) and biomass production. Control: no fertilization; NPK: mineral-only fertilization; M: manure-only fertilization; NPKM: a combination of mineral and manure fertilizers. Bars represent means (*n* = 3) with standard deviations. Letters indicate significant differences (*P* ≤ 0.05) among treatments. Comparisons among treatments were tested by least significant difference post hoc tests.

Compared to the unfertilized soils, microbial CUE in all fertilized soils were higher. Microbial biomass production, which was inferred by DNA concentration changes, increased, especially in manured soils (Fig. 2). Microbial CUE in soils with only mineral fertilization (NPK) increased by 22%, as compared to the soils without fertilization (control). In contrast, manured soils, including those fertilized with only manure (M) and those fertilized with both mineral fertilizers and manure (NPKM), featured an increase of 61% over the control (Fig. 2). The production of microbial biomass in manured soils was >350-ng C/g soil/h, which was three times faster than that of the control and NPK soils (Fig. 2).

### Active bacteria detected by qSIP

**Fig 3:**
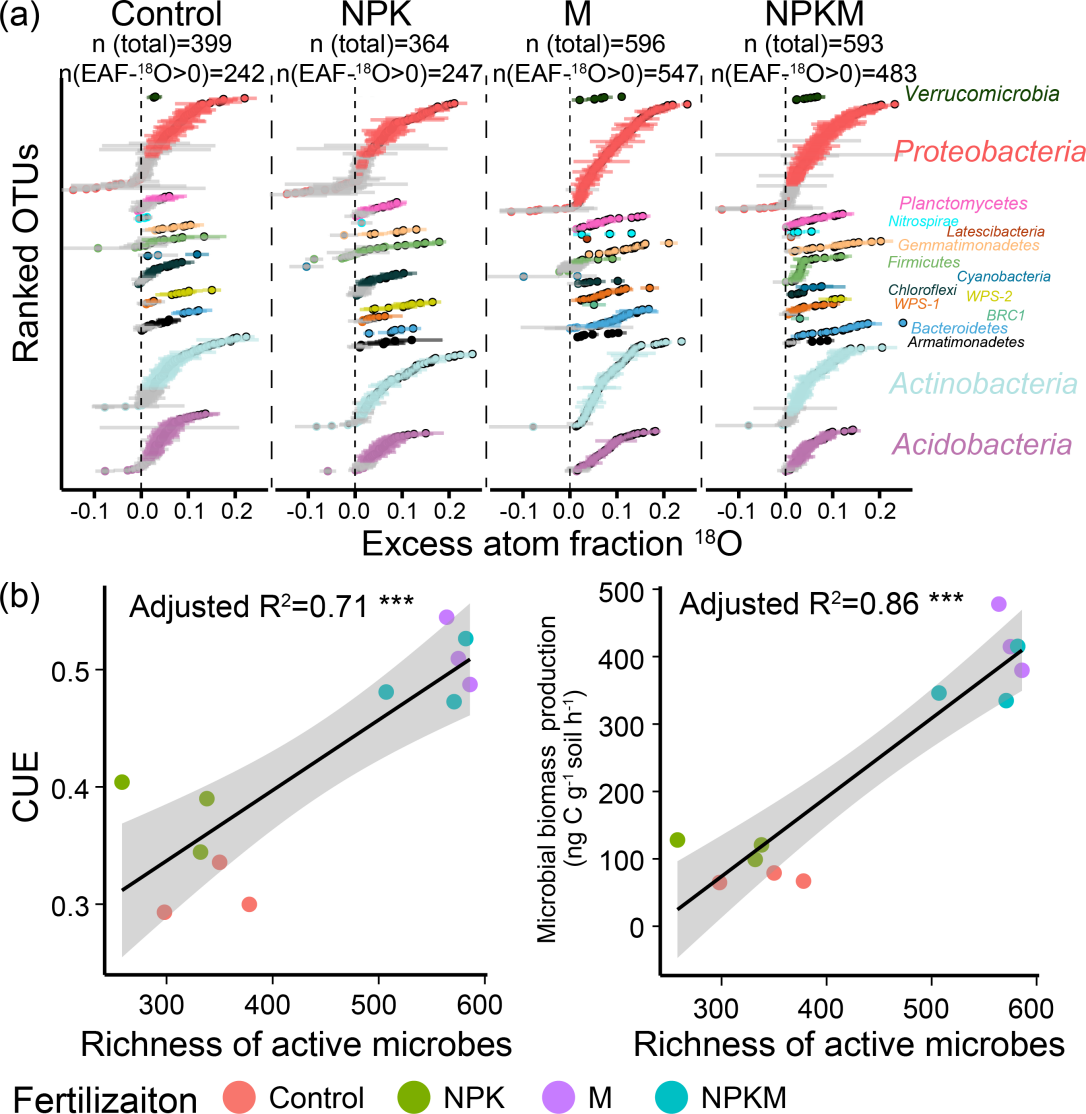
Active microbial communities detected by qSIP. (**a**) OTU-specific shifts in the mean excess atom ^18^O fraction (EAF-^18^O) of OTUs with 95% confidence intervals. The unclassified OTUs at the phylum level were excluded. OTUs are colored by phylum. OTUs without significant ^18^O enrichment are in gray. (**b**) Relationships between microbial CUE/biomass production and the active bacterial richness. *** indicates *P* ≤ 0.001.

Based on qSIP, operational taxonomic units (OTUs) that sufficiently incorporated ^18^O into DNA [lower 95% confidence intervals (CIs) of excess atom ^18^O fraction (EAF-^18^O) >0] were defined as active species (the unclassified OTUs at the phylum level were excluded). The high input of organic resources by manure increased the proportion and richness of active bacteria (Fig. 3a). The control and NPK soils contained 364–399 total OTUs (e.g., OTUs after sequence quality control and prevalence filtering), of which 242–247 OTUs (accounting for 61%–68% of total OTUs) were active (Fig. 3a). Manured soils had a higher richness over that of the control and NPK soils, with 593–596 total OTUs, of which 483–547 were active (accounting for 82%–92% of total OTUs, Fig. 3a). Microbial biomass production and CUE increased, along with the richness of active bacteria (Fig. 3b).

Based on unconstrained principal coordinates analysis (PCoA) ordination, the composition of the active bacterial communities formed distinct clusters according to fertilization type (Fig. S1). Most active species were affiliated with Proteobacteria, Actinobacteria, and Acidobacteria lineages across all soils (Fig. S2a and b). The ^18^O labeling degree of a given species was independent (CK, NPK, and NPKM) or weakly correlated (M, *R*^2^ = 0.03) with its original absolute abundance in the soil microbial reservoir (Fig. S3).

### Bacterial reproduction, mortality, and net growth based on qSIP

**Fig 4:**
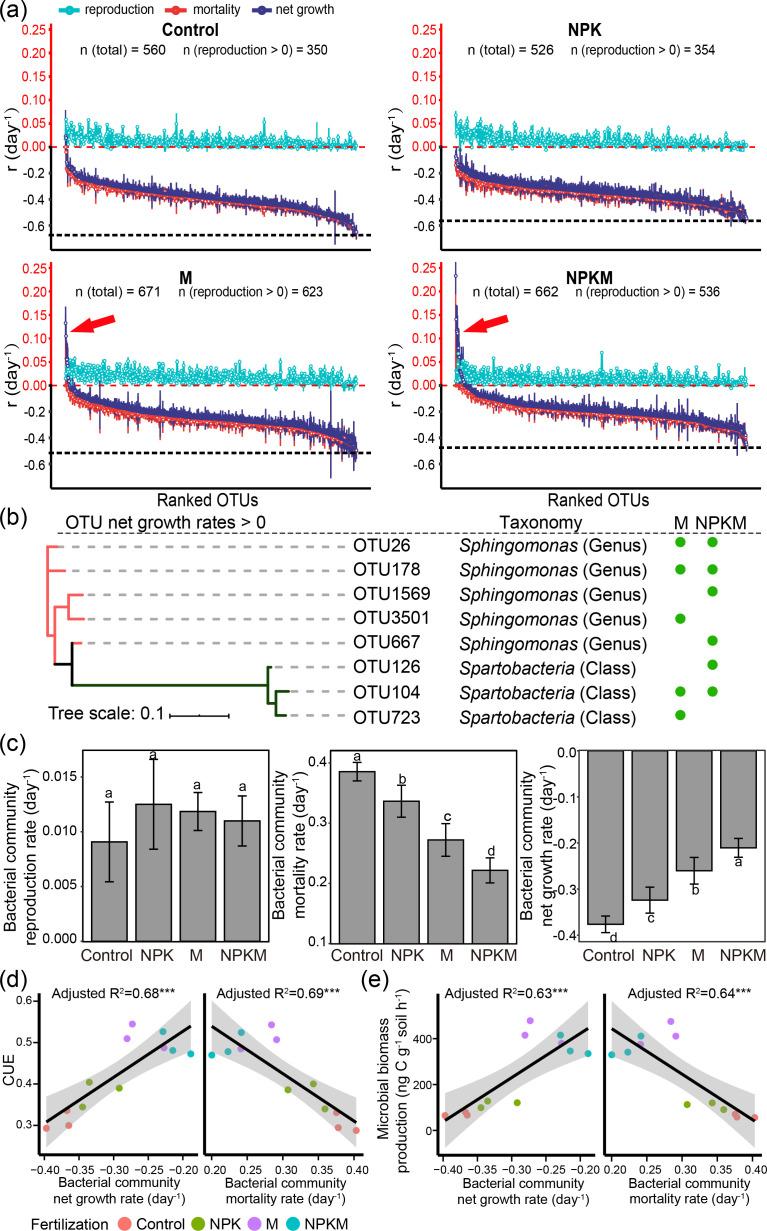
Growth properties of active microbial communities. (**a**) Population growth rates in the control, NPK, M, and NPKM fertilized soils. Nearly all taxa declined in abundance during incubation (net population growth rate <0, dark blue dots), as the mortality rates (red dots) outweighed the rates of reproduction (paler cyan dots). The red arrows point to taxa with net growth rates greater than 0. Dots indicate the means of rates; bars are 95% confidence intervals. OTUs are ranked independently in each panel. Black dashed horizontal lines represent the lowest net population growth rate in each soil. All OTUs were included, including the unclassified OTUs at the phylum level. (**b**) Phylogenetic tree of OTUs with positive net population growth rates. Green dots indicate the presence of OTUs across treatments. (**c**) qSIP-estimated community reproduction rates, mortality rates (mortality is expressed as absolute values for comparison), and net growth rates of soil bacteria. (**d**) Relationships between microbial CUE and bacterial net growth rate/mortality rate. (**e**) Relationships between microbial biomass production and bacterial net growth rate/mortality rate. *** indicates *P* ≤ 0.001.

Compared with the control and NPK soils, manured soils harbored more bacteria with positive reproduction rates (Fig. 4a). Approximately 63%–67% of the taxa exhibited positive reproduction rates in soils without manure addition, whereas the rates increased to 81%–93% in manure-fertilized soils (Fig. 4a). The majority of OTUs (99.6% in control, 99.8% in NPK, 97.2% in M, and 94.7% in NPKM) had negative rates of net growth (Fig. 4a).

Five OTUs in M soils and six OTUs in NPKM soils had positive net growth values. No taxa with a positive net growth rate were detected in control and NPK soils. Seven of the total 11 OTUs were *Sphingomonas* (genus level) of Proteobacteria, and 4 OTUs were classified as *Spartobacteria* (class level) of Verrucomicrobia (Fig. 4b).

Community reproduction rates (i.e., average reproduction rates weighted by abundance) were similar among fertilization treatments (Fig. 4c). Manure addition slowed bacterial community mortality rates by 0.11–0.16/day and accelerated net growth rates by 0.12–0.17/day, as compared to the control and NPK soils (Fig. 4c). Microbial biomass production and CUE increased with the community net growth rate and decreased with the community mortality rate (Fig. 4d and e).

The net growth rate of *Sphingomonas* in manure-fertilized soils increased by 0.18–0.35/day compared to the control and NPK soils (Fig. S4). The absolute abundance of *Sphingomonas* also increased in manure-fertilized soils (Fig. S5).

### Organic carbon metabolisms in soils at community level

**Fig 5:**
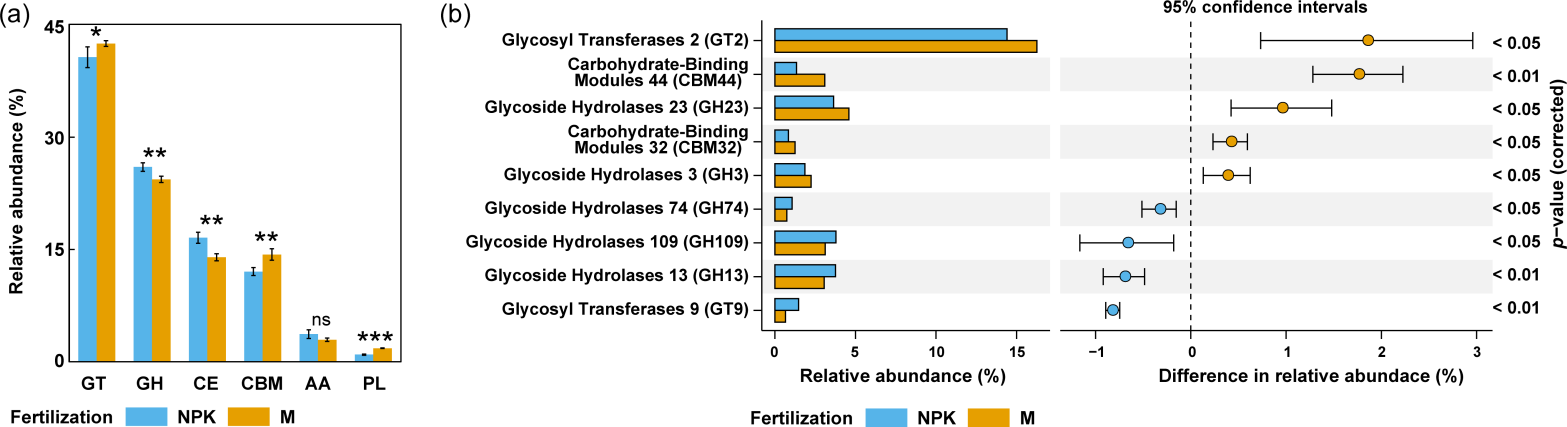
Organic carbon metabolism-related genes (annotated based on CAZY database) at the community level. (**a**) Relative abundances of organic carbon metabolism-related genes at the enzyme class level. “ns” indicates not significant, * indicates *P* ≤ 0.05, ** indicates *P* ≤ 0.01, and *** indicates *P* ≤ 0.001. (**b**) Organic carbon metabolism-related genes with significant differences in abundance at the enzyme family level.

Two representative fertilizer treatments (NPK and M) with significant differences in CUE, MBC production, active bacterial richness, mortality, and net growth rates were selected for metagenomic sequencing in order to explore functions of the active microbial community. We assessed organic C metabolism-related functions based on the CAZY database. For both M and NPK soils, glycosyl transferases (GTs), glycoside hydrolases (GHs), carbohydrate esterases (CEs), and carbohydrate-binding modules (CBMs) were the abundant enzyme classes with relative abundance of >10%, while auxiliary activities, polysaccharide lyase, S-layer homology domain, dockerin domain, and cohesin domain had relatively low abundance (<4%, Fig. 5a; Table S1). The GT functions, which are related to biomass synthesis, exhibited higher abundances in M than in NPK soils. Conversely, the abundances of GH and CE, related to hydrolysis of glycosidic bonds and carbohydrate esters, were higher in NPK soils (Fig. 5a; Table S1).

At the enzyme family level, GH109 (cell wall degradation), GH13 (degradation of starch/oligosaccharides), GT9 (synthesis of membrane and cell wall), and GH74 (degradation of cellulose and hemicellulose) were enriched in NPK soils (Fig. 5b; Table S2). GT2 (synthesis of membrane and cell wall), GH23 (cell wall degradation), CBM44 (binding hemicellulose and cellulose), GH3 (degradation of cellulose and hemicellulose), and CBM32 (binding starch/oligosaccharides and cell wall components) were enriched in M soils (Fig. 5b; Table S2). Alterations in C cycling-related functions were principally driven by members of Actinobacteria and Proteobacteria (Fig. S6a and b).

### Organic carbon metabolisms of *Sphingomonas*

As the only genus with a positive net growth rate detected in this study, the organic carbon metabolisms of *Sphingomonas* were focused on. The results derived from all *Sphingomonas*-annotated contigs showed that the GT functions in *Sphingomonas* had higher abundance in M than in NPK soils, while the abundances of GH and CE in *Sphingomonas* were higher in NPK soils (Fig. S7; Table S3). This indicated that *Sphingomonas* reflected a similar C cycling pattern as compared to the entire community, with a higher abundance of synthesis-related genes but a lower abundance of hydrolysis-related genes in M soils than NPK soils.

At the enzyme family level, GT4 (synthesis of membrane and cell wall), GT28 (synthesis of membrane and cell wall), GH102 (cell wall degradation), GH23 (cell wall degradation), GH43 (degradation of hemicellulose), and CBM50 (binding cell wall components) were enriched in *Sphingomonas* that originated from M soils (Fig. S7; Table S2). In contrast, CE7 (degradation of hemicellulose), GH109 (cell wall degradation), GH1 (degradation of starch/oligosaccharides), and CE1 (degradation of hemicellulose and pectin) were enriched in *Sphingomonas* harbored in NPK soils (Fig. S7; Table S2).

### Metabolic reconstructions of *Sphingomonas*

**Fig 6:**
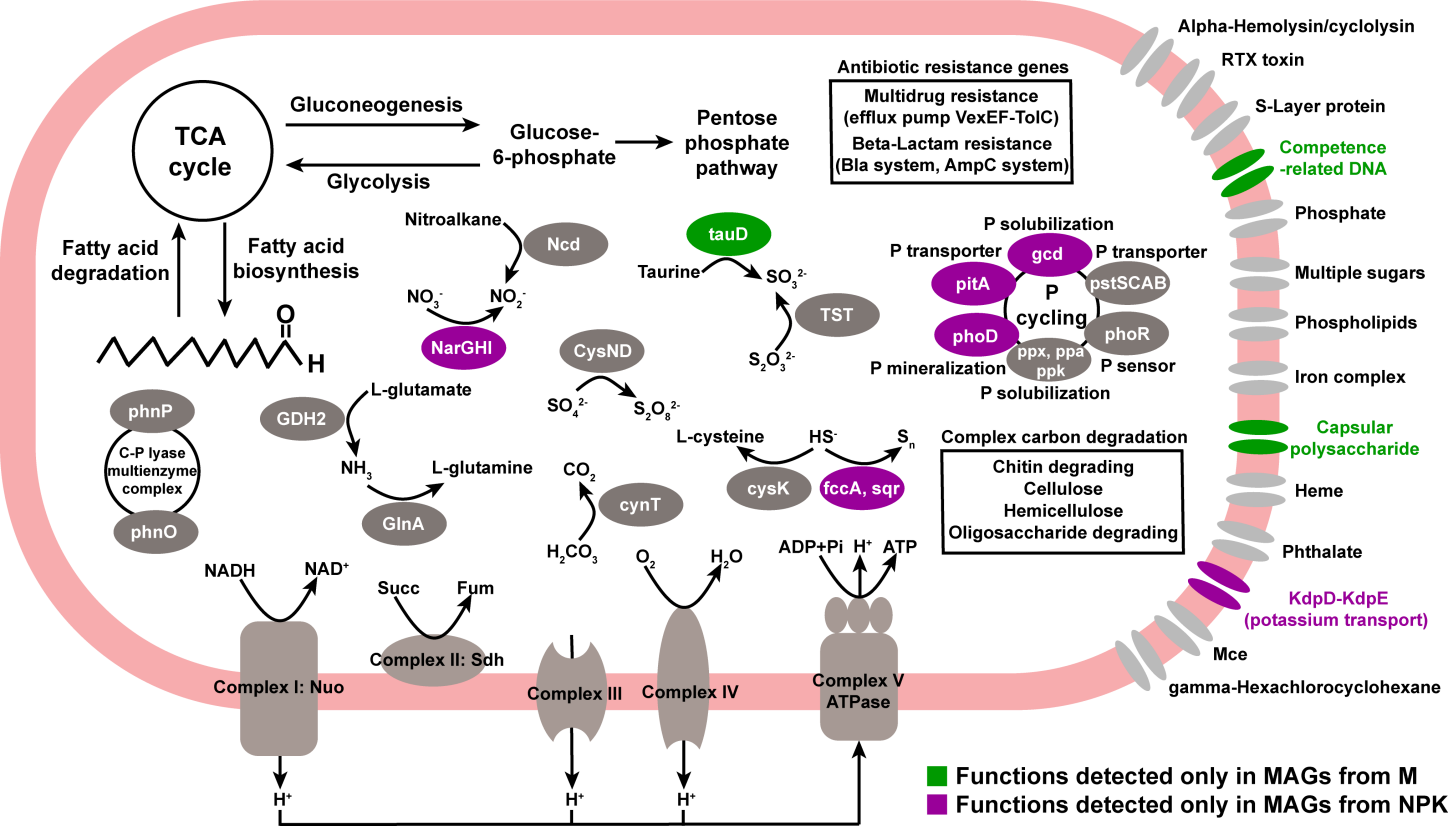
Metabolic reconstructions of *Sphingomonas*.

Four and six metagenome assembled genomes (MAGs; i.e., medium- and high-quality bins with completeness >70% and contamination <10%) of *Sphingomonas* were recovered from M and NPK soils, respectively (Fig. S8). Genomic analyses indicated that these *Sphingomonas* MAGs possess multiple functions related to the metabolism of C, nitrogen, phosphorus, and sulfur (Fig. 6). *Sphingomonas* MAGs exhibited central C metabolism (e.g., (tricarboxylic acid) TCA cycle, gluconeogenesis, pentose phosphate pathway, and fatty acid degradation) and ability for the degradation of complex organic compounds (e.g., chitin, cellulose, hemicellulose, and oligosaccharide degradation), ensuring various pathways for C utilization during growth (Fig. 6). Oxidative phosphorylation complexes involved in energy conservation were also detected. In addition, antibiotic resistance, including multidrug resistance (efflux pump *VexEF*-*TolC*) and beta-lactam resistance (*bla* and *ampC* system), may improve the tolerance of *Sphingomonas* to biotic stress.

Most functions were shared by *Sphingomonas* MAGs in both M and NPK soils such as nitrogen metabolism (*GDH2*, *GlnA*, and *Ncd* genes), sulfur metabolism (*TST*, *cysK*, and *CysND* genes), phosphorus metabolism (*ppx*, *ppa*, *ppk*, *phoR*, and *pstSCAB* genes) and transport systems (e.g., multiple sugar, phospholipid, and phthalate). However, there were some cell processes detected only in *Sphingomonas* MAGs from specific soils (Fig. 6). For example, *Sphingomonas* MAGs from M soils had unique functions related to the transport of capsular polysaccharide and competence-related DNA and a unique *tauD* gene (taurine dioxygenase). In NPK soils, *Sphingomonas* MAGs contained unique functions related to potassium transport (*KdpD*-*KdpE* system), phosphorus solubilization (*gcd* gene), phosphorus transporter (*pitA* gene), phosphorus mineralization (*phoD* gene), nitrate reduction (*NarGHI* gene), and sulfide oxidation (*fccA* and *sqr* gene).

Furthermore, 24 *Sphingomonas* clones were isolated from soils and incubated in the sterilized soil supernatant of M and NPK soils (Fig. S9). About 55% and 50% *Sphingomonas* cells were active in M and NPK soil supernatants, respectively. Of 24 *Sphingomonas* isolates, 18 had more active cells when incubated in the M soil supernatant than in the NPK soil supernatant. Compared with NPK soil supernatant, 87.5% *Sphingomonas* isolates produced more biomass (i.e., higher cell count) with an average increase from 189 to 228 cells/µL in M soil supernatant. About 79% *Sphingomonas* isolates increased CUE in the M soil supernatant than in the NPK soil supernatant, with an average increase of 10.6%.

### Other parameters reflecting microbial life strategies

Based on the metagenomic data, additional parameters (i.e., average genome size, codon usage bias, and maximum growth rate) were calculated to reflect microbial life strategies. A larger average genome size was found in M soils (Fig. S10), suggesting a higher resistance of soil microbes to environmental disturbances. Microbes in M soils have a greater codon usage bias in their ribosomal genes (Fig. S10), indicating fast growth, since microbes increase codon usage bias when undergoing translational selection during fast growth. This is also supported by the higher predicted maximum growth rate in M soils (Fig. S10).

**Fig 7:**
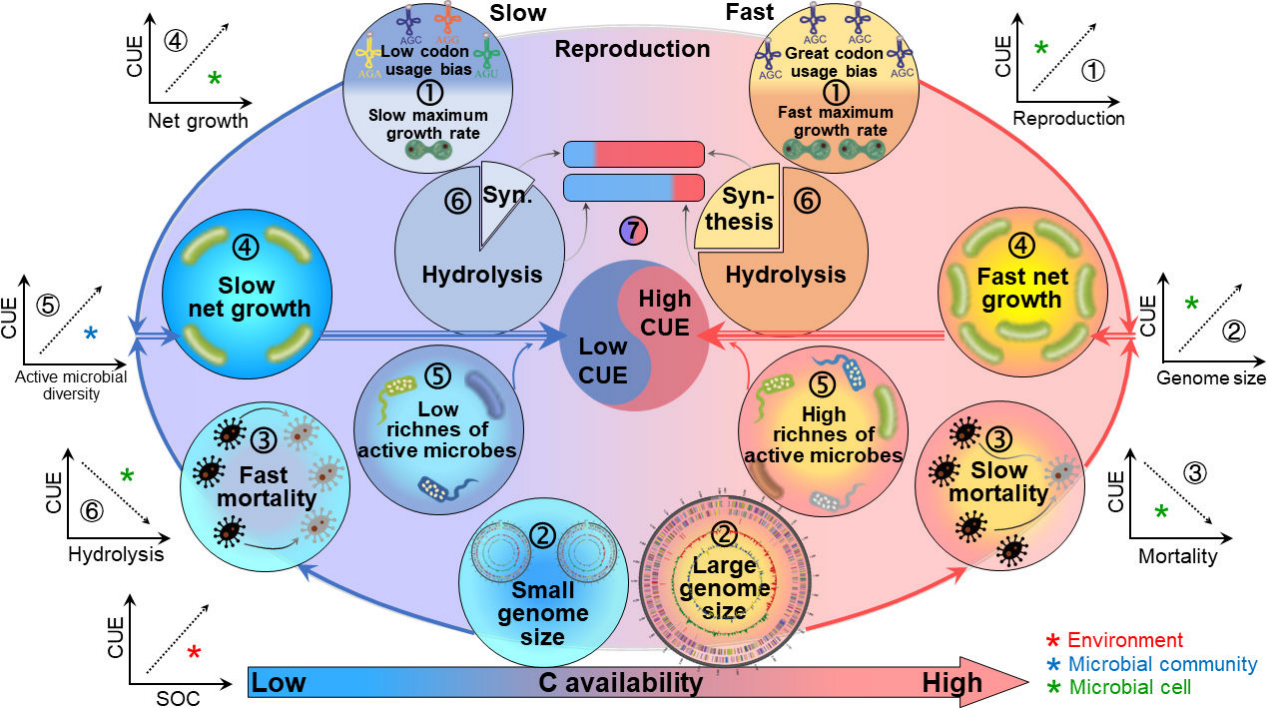
Conceptual figure illustrating microbial population dynamics, life strategies, and their impacts on carbon use efficiency (CUE). In soils with higher carbon availability, (**i**) greater codon usage bias and faster predicated maximum growth rates indicate faster reproduction rates. (ii) Larger genome sizes may imply (iii) slower mortality. Faster reproduction and slower mortality result in (iv) higher net growth. Higher carbon availability also results in (v) a higher richness of active microbes and (vi) higher abundances of biomass synthesis-related genes. These changes in population dynamics and life strategies explain the (vii) higher microbial CUE in soils with high carbon availability. The asterisks of different colors represent distinct types of factors influencing CUE: red asterisks indicate environmental variables; blue asterisks indicate properties of microbial communities, i.e., community-level traits; and green asterisks indicate properties of individual microbial cells, i.e., cellular traits. In the context of our results, the term “fast net growth” reflects a relative increase in the net growth rate. It does not imply rapid absolute growth but rather a reduced rate of decline in the population size.

Overall, microbes in organically amended soils with high SOM have greater codon usage bias and higher predicated maximum growth rates, which indicate higher reproduction rates. Mortality rates were slower, possibly resulting from a larger genome, and thus, the corresponding higher net growth rates. Soils with high SOM also resulted in a higher richness of active microbes and a larger abundance of biomass synthesis-related genes. These changes in population dynamics and life strategies may explain the higher microbial CUE in soils with high SOM (Fig. 7).

## DISCUSSION

Increasing C sequestration in soils is an important pathway that can be harnessed to mitigate global climate change by reducing greenhouse gas emissions from soil and storing photosynthetically derived C. SOC in arable soils is the core of soil fertility that ensures crop production and food security. Agricultural practices can influence the composition and functions of soil microbial communities, thus altering microbially mediated C cycling. Deeper studies on the processes that underlie the microbially mediated transformations and sequestration of soil C are important issues that must be addressed in the context of soil and environmental science. We found that long-term organically amended soils, which have a high accumulation of SOM, had a high potential for the sequestration of microbially derived C (i.e., high CUE; Fig. 2). Organic fertilization may be a driving factor that supports active microbes that possess specific population dynamics and life strategies, such as fast net growth rates (Fig. 4) and anabolic-biased C cycling pathways (Fig. 5).

### High SOM content supports elevated microbial CUE

Living microbes produce cell polymers from organic compounds and, upon death, contribute to SOM in the form of resistant microbial necromass. We found that the CUE and microbial biomass production in organically fertilized soils were larger than that in soils amended with mineral fertilizers (Fig. 2). While it is important to consider the biomass-to-necromass conversion rate, when assessing overall carbon sequestration potential, it is generally desirable to generate more microbial biomass. This is because the increase in microbial biomass will ultimately lead to the formation of additional necromass (24, 25), which is an important contributor to stable SOC pools (1, 26). In this context, additional C input by manure may be a key factor that influences microbial CUE. By integrating the results of published studies that investigated microbial CUE using the ^18^O-H_2_O method, we identified a positive linear relationship between SOC content and microbial CUE (Fig. S11). This supports the universality of our findings in soils, at least within the range of SOC up to 50 g/kg, which covers the SOC content of most farmland soils and many natural soils. This finding is also supported by the recent publication (7). However, several studies have shown that when the soil C content exceeds a threshold, microbes tend to release energy (energy spilling), and the microbial CUE declines with further increase of the C content (27–29). However, the C return in farmland soils is limited with the availability of organics not always sufficient to satisfy the microbial demand. Thus, the increase in C induced by organic fertilization stimulates microbial growth and, consequently, results in a high CUE, rather than causing an energy “spilling” effect.

It has also been reported that high soil SOM increases plant growth, resulting in additional C inputs into soils through rhizodeposition (30). The plant and microbe mediation of C sequestration might have an additive effect: the more fertile farmland soil becoming enriched in organic C. Therefore, organic fertilization is an effective practice not only to increase yield but also to increase soil C storage to achieve a “carbon neutrality” strategic goal.

### A relative increase in net growth rate in organically amended soils improves microbial CUE

The CUE is strongly driven by microbial community diversity and composition by regulating metabolic capacity and functions (31, 32). In soils, most microorganisms are in a dormant state and have low activity (33, 34). Organic amendment provides a multitude of diverse and accessible C resources (10, 12), thus boosting microbial richness and activity in soil (12). This is consistent with the increased richness and proportion of active bacteria identified in organically amended soils (Fig. 3). Under this condition, the higher biodiversity would be expected to allow the microbial community to occupy a wider niche and utilize a wider spectrum of resources (35), as well as increase the probability of complementary interactions between species via cross-feeding and syntrophy (32). This results in a more productive community with more complementary and efficient resource utilization (32, 36) and thus an increased CUE.

A recent investigation has demonstrated that microbial growth rates are correlated to CUE (37), indicating that population dynamics (e.g., mortality and growth rate) may help drive changes in CUE. Bacterial mortality rates were reduced in organically fertilized soils as compared to soils amended with mineral fertilization (Fig. 4c). Furthermore, the CUE increased with reduced bacterial mortality rate (Fig. 4d). This is because greater biodiversity induced by organic fertilization increased resource use efficiency and interactions among species (35), which lowered the possibility of mortality. The larger average genome size (Fig. S10) might explain the slower microbial mortality rate in manured soils. A larger genome size implies increased functional complexity, which facilitates regulations in response to environmental disturbances. Microorganisms with larger genome sizes would be expected to also have a higher resistance to environmental stress (38), since they have more candidate resistance genes to draw upon. However, it’s essential to also consider the trade-off between enhanced functional capacity and the energetic costs associated with maintaining larger genomes. Larger genomes typically require more resources for maintenance, which could paradoxically lead to higher intrinsic mortality rates and lower CUE under certain conditions.

The lower community mortality rate identified in organically fertilized soils might suggest a longer average microbial life span. For example, the community mortality rate in M soils was 0.27/day with an average active microbial lifespan of 3.7 days (i.e., the multiplicative inverse of the mortality rate). As an important physiological parameter, microbial life span may serve to regulate the physiological states of cells, such as cell size, generation time, and stress tolerance (39). For example, the prolonged life span of *Escherichia coli* by genetic manipulation was found to enlarge cell sizes and increase metabolite production (40). Thus, a longer life span may benefit the production of microbial biomass. A prolonged life span also implies increased cell vitality and a higher possibility of replication (41), which increases microbial net growth. In energy-limited environments, an extended microbial life span indicates efficient C utilization and maximum fitness (42). Thus, the prolonged life span of microbes under long-term organically fertilized soils may be a crucial trait that increases microbial CUE. However, we should note that using the inverse of mortality rates as a proxy for microbial life span does not constitute a direct measure of life span and may oversimplify the complexity of microbial life spans. The life span estimates derived from our study could be influenced by specific experimental conditions, such as the re-wetting event associated with qSIP and the relatively short incubation period. Further research with direct life span measurements would provide validation and deeper insights into our findings.

The water addition to soils often triggers high mortality in bacterial communities (43, 44). Specifically, the re-wetting of seasonally dry soils can cause immediate and significant bacterial mortality—up to 25% gene copy loss within just 3 h. This phenomenon is likely due to the lysis of living cells caused by rehydration stress (45) and the rapid degradation of dead cells (46). In our study, the estimated bacterial community mortality rates ranged from 0.22 to 0.39/day (Fig. 4c), which aligns with community-level turnover rates observed in a previous qSIP study (43). To further validate these findings, we conducted a quantitative PCR (qPCR) analysis on soil DNA before and after the incubation. The results revealed a decrease in the total bacterial copies across all treatments after incubation (Fig. S12), supporting the observed high mortality rates (Fig. 4c). In addition, the observation of high mortality rates and incorporation of ^18^O into DNA are not necessarily contradictory. The incorporation of ^18^O indicates active microbial reproduction, as it reflects the synthesis of new DNA in living cells. High mortality rates can co-occur with high reproduction rates in microbial populations. This phenomenon can be attributed to the rapid turnover of microbes, where a significant proportion of the population is actively growing and incorporating ^18^O, while another part is undergoing cell death. This rapid turnover is a characteristic feature of many microbial communities, especially under conditions that stimulate growth, such as the re-wetting event (47).

Organic amendment increased the net growth rate of the community, which had positive impacts on microbial CUE (Fig. 4c and d). This indicates that the growth rate-yield trade-off (negative relationship between growth rate and microbial CUE) was not found in farmland soils. This trade-off is the consequence of balancing the speed and accuracy of translation (48, 49). However, microbial CUE is not absolutely determined by the growth rate-yield trade-off; it can also be affected by the rate of substrate uptake (50). In resource-limited farmland soils, the microbial growth rate is slower as compared to a laboratory culture, and less energy is used to correct translation errors due to fast growth. Thus, the occurrence of a growth rate-yield trade-off is condition dependent and is supposed to have limited effects in this study, whereas the relative increase in net growth rate caused by organic amendment increased the microbial CUE.

### Energy allocation to resource acquisition and stress tolerance is the curial trait determining microbial CUE

In addition to the above-mentioned population dynamics, the trade-off in energy allocation between growth and survival (e.g., maintenance, nutrient acquisition, and stress tolerance) is an important life strategy that influences the microbial CUE. The abundance of genes involved in hydrolysis of glycosidic bonds and carbohydrate esters was higher at both the community and species levels in mineral-only fertilized soils than in organically fertilized soils (Fig. 5; Fig. S7). In mineral-only fertilized soils with lower C content, microorganisms have to invest more energy to produce diverse extracellular enzymes to depolymerize organic compounds for energy and nutrient acquisition (21, 23, 49). However, in organically fertilized soils with high C availability, the lower nutrient-acquisition cost resulted in more C allocated for growth and thus an increased microbial CUE.

A favorable stoichiometric balance is another potential driver that supports higher CUE in organically fertilized soils as compared to those under mineral fertilization. Microbes have to maintain their elemental composition within a narrow range (51). The C:N ratio in SOM is lower than the optimum ratio for microbial growth (52). Long-term mineral fertilization increased the stoichiometric imbalance (low C: N and C: P ratios), while C, N, *P* and other nutrients introduced by organic fertilization create a more suitable environment for microbial growth (9). When living in organically fertilized soils with favorable stoichiometry, microorganisms allocate less energy to maintain microbial C:nutrient balance than in mineral fertilized soils (3, 4, 53). Furthermore, organic fertilization alleviated soil acidification induced by mineral fertilization (Table S4), indicating reduced energy cost for stress tolerance and increased growth yield (49).

### The implications of fast net growth rate in *Sphingomonas*

Remarkably, although various microbial taxa contribute to energy and matter fluxes in soils (Fig. 3a), *Sphingomonas* was the only genus identified with positive net growth (Fig. 4b). As a usual inhabitant in cow gut, this growth advantage of *Sphingomonas* might be contributed by manure addition. *Sphingomonas* is characterized by its high mineral-weathering potential (54), intensive soil organic matter decomposition (55), and plant growth promotion (56). Plants can recruit *Sphingomonas* to enhance their ability to defend against pathogens (57, 58). These functions support a generalist role with universal distribution. By recovering genomes from metagenomic data, we found that *Sphingomonas* possessed diverse functions, including multiple nutrient metabolism (e.g., C, N, P, and S), complex C degradation (e.g., chitin, cellulose, hemicellulose, and oligosaccharide), and antibiotic resistance (Fig. 6). These characteristics indicate that *Sphingomonas* is likely a strong competitor in these farmland soils. While there were also differences between genomes recovered from organically and mineral fertilized soils (Fig. 6), we did not find direct evidence at the genome level that could explain the higher net growth rate of *Sphingomonas* in organically fertilized soils. The measured net growth rate difference of *Sphingomonas* may be due to an overall more supportive soil environment (higher nutrient availability and neutral pH, Table S4) under organic fertilization, which increases the survival of *Sphingomonas* and boosts its abundance (59). This is partly proved by our results that *Sphingomonas* had more active cells, higher biomass, and CUE when growing in the sterilized soil supernatant of M soils than NPK soils (Fig. S9).

With its functional potential for plant growth promotion and growth advantage, *Sphingomonas* may be a candidate inoculant that could support both an increase in SOM and crop yield. The observed characteristics of *Sphingomonas* in this study, which demonstrated a relatively higher net growth rate than other taxa, serves as an example that different microbial taxa may have individualistic effects on community-level heterotrophic growth and respiration. Thus, future efforts should also be devoted to evaluating the contributions of individual bacterial taxa to C fluxes in soil. This study primarily focused on bacterial contributions to microbial CUE, yet it is important to note that community-level CUE includes a range of microorganisms, particularly fungi. Considering the differing CUE between bacteria and fungi (60), future research should include a thorough examination of fungi to better understand soil-atmosphere carbon exchange dynamics.

Soil microorganisms perform dichotomous roles in the mineralization and stabilization of SOC. The balance between these two processes, as reflected by CUE, determines the extent of microbially mediated C sequestration in soils. Long-term fertilization provides habitats for microbes with specific ecological strategies that impact the CUE. Herein, we found that soils with large organic matter contents that accumulated under long-term organic fertilization maintained higher microbial CUE than soils with low SOM. This indicates that more fertile soils will become richer in organic C through a more efficient microbial utilization of resources. The organic amendment creates suitable environments with high C availability, microbe-favoring C:N:P stoichiometries, and a neutral soil pH. Consequently, microorganisms invest less energy and resources for C and nutrient acquisition as well as stress tolerance. These optimal soil conditions support a more diverse active microbial community characterized by accelerated net growth rate and anabolic-biased C cycling. Under these conditions, microbes produce more microbial biomass and, later, more necromass, which benefits stable SOM pools. Our study demonstrates how microbially mediated C sequestration is influenced by microbial population dynamics and life strategies, which, in turn, will be useful for harnessing soil capabilities to mitigate further impacts of climate change.

## MATERIALS AND METHODS

### Site description and soil sampling

Soils were sampled from a long-term fertilization experiment established in 1986 and located in Jiangxi province, China (116°33′E, 28°25′N). There were four fertilization regimes: (i) no fertilizer (control soil); (ii) mineral-only fertilizers (NPK soil); (iii) manure-only fertilizers (M soil); and (iv) mineral fertilizers combined with manure (NPKM soil). Each treatment contains three field replicates. The topsoil (0–20 cm) was collected for further study. Soil properties were provided in Table S4, and the detailed field experiment designs were provided in a previous study (9). Considering the inherent influence of the microbial community present in the manure fertilizer, the soil samples were collected more than 4 months after manure application. This extended period surpasses the typical life span of externally introduced microbes in a new natural environment, particularly in soils with high microbial diversity. Such a sampling strategy ensures that our findings indicate the long-term effects of the manure addition, rather than immediate alterations from the manure’s native microbial population.

### CUE measurement using ^18^O-H_2_O method

Microbial CUE was determined based on the incorporation of ^18^O into DNA using the ^18^O-H_2_O incubation method (19). Soils were pre-incubated (adjusted to approximately 20% moisture content) at 25°C in the dark for 6 days and then air-dried on clean sterile trays for 24 h at room temperature to largely avoid the influence of the original abundance of naturel (H_2_^16^O) water. For each soil sample, 2-g dry soil was incubated with 400 µL of 98 atom% H_2_^18^O in the dark at 25°C. To estimate the amount of CO_2_-C production (C_respiration_), after 24-h incubation, the CO_2_ production in the microcosm was determined using a gas chromatograph (Agilent 7890, Santa Clara, CA, USA). Concurrently, in order to determine the production of microbial biomass C (C_growth_), the DNA of ^18^O-H_2_O labeled soils was extracted using a FastDNA SPIN Kit for Soil (MP Biomedicals, Cleveland, OH, USA). The concentration of extracted DNA was determined fluorometrically using a Qubit DNA HS (high sensitivity) assay kits (Thermo Scientific, Waltham, MA, USA) on a Qubit 4 fluorometer (Thermo Scientific). DNA was dried in silver capsules at 60°C overnight (about 10 h), and the ^18^O abundance and total O content were measured using a thermochemical elemental analyzer coupled with a Conflo III open split system to an isotope ratio mass spectrometer (TC/EA-IRMS, Delta V Advantage; Thermo Fisher, Germany). Parallel microcosms with H_2_^16^O were also prepared for each treatment and followed the same experimental processes. The amount of DNA produced was calculated based on the abundance of ^18^O in the labeled DNA, the non-labeled DNA (natural abundance), and the soil water of the labeled sample (61). The C_growth_ was then calculated based on the microbial biomass C (MBC) produced during incubation. We inferred MBC production from the amount of DNA produced using a sample-specific conversion factor (32). This factor was used to correlate the concentration of soil DNA and the total soil MBC, enabling the conversion of DNA production into an equivalent MBC production (Table S5). Based on steady-state assumptions, the C taken up by microbes (C_uptake_) was the sum of C_respiration_ and C_growth_; thus, microbial CUE was calculated by the following equation: CUE = C_growth_ / C_uptake_.

### Isopycnic ultracentrifugation, fraction, and qPCR

The isopycnic ultracentrifugation and fraction of DNA from both ^18^O-H_2_O and ^16^O-H_2_O added soils were performed according to a reported protocol (62) with some modifications. Briefly, 3-µg DNA was added into 1.85-g/mL CsCl gradient buffer (0.1-M Tris-HCl, 0.1-M KCl, 1-mM EDTA, pH = 8.0) with an initial CsCl buoyant density of 1.718 g/mL. Density gradient centrifugation was performed in 5.1-mL Quick‐Seal polyallomer ultracentrifugation tubes (Beckman Coulter, Palo Alto, CA, USA) in a VTi 65.2 vertical rotor (Beckman Coulter) and subjected to centrifugation at 177,000 g for 72 h at 18°C. Centrifuged gradients were fractionated into 20 equal volumes (~250 µL each, 500 µL/min) by displacing the gradient medium with sterile water at the top of the tube using a syringe pump (Longer Pump, LSP01‐1A, China). Next, 30-µL aliquots were used to measure the refractive index with an AR200 digital refractometer (Reichert, Inc., Buffalo, NY) in order to determine the buoyant density of each collected fraction. Fractionated DNA was precipitated from CsCl by adding 500 µL 30% polyethylene glycol (PEG) 6000 and 1.6-M NaCl solution, incubated for 1 h at 37°C, and then washed twice with 70% ethanol. The samples were then dissolved in 30 µL of Tris‐EDTA buffer.

qPCR was employed to determine the abundances of bacterial 16S rRNA genes in total soil DNA at day 0, total soil DNA after incubation, and fractionated DNA in each density fraction. Standard curves were generated using 10‐fold serial dilutions with primers 515F (5′‐GTG CCA GCM GCC GCG G‐3′) and 907R (5′‐CCG TCA ATT CMT TTR AGT TT‐3′); the fragment amplified was 392 bp. Each reaction was performed in a 25-µL volume containing 12.5-µL SYBR Premix Ex Taq (TaKaRa Biotechnology, Otsu, Shiga, Japan), 0.5 µL of each primer (10 µM), 0.5 µL of ROX Reference Dye II (50×), 1 µL of DNA template, and 10.5 µL of sterile water. The amplification conditions were as follows: 95°C for 5 min, 40 cycles of 15 s at 95°C, 34 s at 64°C, and a final temperature increase to 95°C for 15 s.

We further determined the recovery efficiencies for each fraction and integrated these values into qSIP analyses. To calculate the recovery efficiency, the sum of gene copy numbers of each fraction (weighted by the corresponding volume) within the sample was compared to the gene copy number of unfractionated DNA after incubation. The adjusted gene copy number for each fraction was obtained by dividing the initial gene copy number of that fraction by its calculated recovery efficiency.

### Amplicon sequencing and active microbes detecting

DNA from 0-day pre-wet samples (unfractionated) and fractionated samples with densities between 1.69 and 1.74 g/mL (samples not in this density range had almost no DNA revealed by qPCR, and the proportion of 16S rRNA gene abundance in selected fractions accounted for more than 99.9% of the total 16S rRNA gene abundance; Fig. S13) were used for high-throughput sequencing targeting the 16S rRNA V4-5 region (515F/907R). Sequencing was performed on an Illumina MiSeq (2 × 250 bp) platform. Sequencing data were processed following the UPARSE pipeline (63). Briefly, the forward and reverse reads were merged, and reads with a quality score less than 30 or a length less than 370 bp were filtered. Singletons were discarded. Sequences were clustered into OTUs at a 97% threshold. Taxonomic identification was performed using the RDP classifier with a threshold of 0.8 (64). We discarded any OTUs that accounted for <0.005% of the total sequences (43) and OTUs that were not present in at least two of the three replicates were removed.

EAF-^18^O, as well as per capita reproduction, mortality, and net growth rates, was calculated using the “qSIP” package in R (37). The buoyant density of each OTU was weighted based on its relative abundance in each fraction. The molecular weights of the DNA were then calculated for each OTU from both unlabeled and labeled samples, which were used to estimate the per-OTU shift in buoyant density due to isotope incorporation. The population dynamics estimation required measuring 16S rRNA gene abundance at the start and end of an incubation, along with changes in ^18^O composition of these genes for each taxon. The calculations are based on the premise that DNA replication rate directly reflects the growth rate of dividing cells. The approach also assumes that a consistent proportion of oxygen in newly synthesized DNA originates from water (43, 62). The calculations quantitatively link increases in the abundance of 16S rRNA gene to growth, while decreases in this gene indicate mortality. By employing an exponential growth model, we estimate per capita reproduction rate from the rate of ^18^O-labeled 16S rRNA gene emergence, and per capita mortality rate from the loss of unlabeled 16S rRNA genes (43). The net growth rate is determined by balancing the reproduction rate and mortality rate. Bootstrapping with re-sampling of replicates within each treatment was used to generate uncertainty estimates of 95% CIs around the per-taxon EAF, reproduction, mortality, and net growth rates. An OTU was considered to show isotopic incorporation (true positive) if the lower 95% CIs of its EAF value were >0; if so, then the OTU was considered active.

This qSIP method, involving the drying of soils and subsequent addition of ^18^O-H_2_O, simulates a re-wetting event. This might lead to higher estimates of microbial growth and respiration compared to undisturbed soil conditions (47). Although these estimates might not precisely reflect field conditions, this approach is closer to *in situ* conditions than traditional lab cultures. Importantly, it has the advantage of resolving rates of reproduction and mortality for individual taxa (43). When estimating population dynamics, we assumed that both unlabeled and labeled 16S rRNA gene copies are lost at the same rate due to cell death during the incubation with ^18^O-labeled water, and that all newly synthesized 16S rRNA gene copies were labeled (43). This approach, while potentially not capturing the full extent of microbial mortality through the degradation of unlabeled 16S rRNA genes, is consistent with previous studies that utilized radiolabeling techniques with thymidine or leucine (43). In addition, our calculated absolute abundances of microbial populations are approximations (43), impacted by challenges such as incomplete DNA extraction from soil cells (65), variations in 16S rRNA gene copies among taxa, and biases in amplification and sequencing (66, 67). Additionally, the qSIP, which assumes the use of oxygen from water for DNA synthesis, may lead to underestimation of reproduction rates. This could occur if nucleotide recycling happens or if new formed cells incorporate pre-existing, unlabeled nucleotides (43). Despite these potential limitations, qSIP provides a consistent framework for estimating microbial population dynamics.

A phylogenetic tree of active microbes based on amplicon sequencing was constructed based on FastTree and visualized using iTOL. General linear regression analysis was used to assess the strength and significance of the correlations. Comparisons among treatments were tested by least significant difference post hoc tests.

### Metagenomic sequencing and genome binning

Two representative treatments (NPK and M) with significant differences in CUE, MBC production, active bacterial richness, and mortality and net growth rates, were selected for metagenomic sequencing to explore the effects of fertilization on microbial CUE at functional and genome levels. The “heavy” fractions (gray area in Fig. S13), where ^18^O-labeled fractions had significantly higher 16S rRNA gene abundances as compared to the ^16^O-labeled fractions, were concentrated and purified for metagenomic sequencing to reconstruct active microbial ecological processes. In these heavy fractions, there was no overlap between ^18^O-labeled and ^16^O-labeled fractions since the 16S rRNA gene abundances in the ^16^O-labeled fractions within the selected fractions were almost 0 (gray area in Fig. S13). Hence, the DNA in heavy fractions of ^18^O-labeled treatments representatively indicated the active microbial ecological processes (68). We conducted three replicates for each treatment during the collection and metagenomic sequencing of the heavy fractions. For each of these replicates, all the heavy fractions (comprising four fractions as indicated by the gray area in Fig. S13) were pooled into a single sample for sequencing. Paired-end sequencing was performed on an Illumina NovaSeq 6000 sequencing platform, yielding reads of ~150 bp. Approximately 240-G raw data were generated. Low-quality reads and adapter sequences were filtered using Trimmomatic v.0.38 (adaptor trimming, average quality = 20). MEGAHIT v.1.1.3 was used to assemble reads into contigs. Prodigal v.2.6.1 was used for predicting open reading frames (ORFs). To estimate the functions related to C metabolic pathways, the ORFs were annotated against the Carbohydrate-Active enZYmes Database (CAZY database). Contigs were also clustered to recover MAGs using MetaBAT v.2. CheckM v.1.1.3 was used to evaluate the completeness and contamination of MAGs. Low-quality MAGs (completeness <70% and contamination >10%) were filtered. The taxonomic annotation and phylogenetic tree were constructed based on the Genome Taxonomy database (GTDB) using GTDB-Tk v.1.5.0. CoverM v.0.5.0 was used to estimate the abundance of MAGs across samples. The connection between *Sphingomonas* MAGs and OTUs was established at the genus level due to challenges in extracting sufficient 16S rRNA sequences from MAGs for phylogenetic analysis. This approach might not accurately reflect the precise phylogenetic identity or nucleotide similarity between the active taxa identified through qSIP and those represented in the MAGs.

Average genome size, codon usage bias, and maximum growth rates of the active communities were calculated based on quality-controlled reads and assembled contigs from metagenomic data in order to explore the microbial life strategies across different treatments (69). Metabolic reconstructions of MAGs were conducted using the Reconstruct Pathway tool in KEGG (https://www.genome.jp/kegg/mapper.html).

### Fluorescence staining and flow cytometry analysis

As *Sphingomonas* was the only genus identified with positive net growth in this study, we took it as an exemplary species for an auxiliary experiment. We isolated bacteria from M and NPK soils, identifying different species within the genus *Sphingomonas* through sequencing of the full-length 16S rRNA gene. These *Sphingomonas* species were then incubated in the sterilized soil supernatants of M and NPK soils. Cell count and activity estimation were conducted using flow cytometry on the Beckman Coulter CytoFLEX platform. After staining with the SYBR Green I and propidium iodide (Invitrogen, USA), live cells emit green fluorescence and dead ones emit red fluorescence (70). Green (SYBR Green I) fluorescence was collected in the FITC channel at 525 nm, and red (PI) fluorescence was collected in the PC5.5 channel at 610 nm. The *t*-test was used to compare parameters between NPK and M, with *P* values adjusted by false discovery rate correction if necessary.

## Data Availability

The amplicon and metagenomics sequences obtained in this study have been submitted to the National Center for Biotechnology Information Sequence Read Archive (http://www.ncbi.nlm.nih.gov/Traces/sra/) under accession number SRP374627 and SRP374859, respectively. The related codes are available at https://github.com/LingLi-AnalysisCode/microbial-regulation-of-carbon-use-efficiency.

## SUPPLEMENTAL MATERIAL

Supplemental material mbio.00177-24-s0001.docx Supplemental tables and figures.

ASM does not own the copyrights to Supplemental Material that may be linked to, or accessed through, an article. The authors have granted ASM a non-exclusive, world-wide license to publish the Supplemental Material files. Please contact the corresponding author directly for reuse.
