# Supplementary material for: Active microbial population dynamics and life strategies drive the enhanced carbon use efficiency in high-organic matter soils
Source: mBio. 2024 Feb 20;15(3):e00177-24. doi: 10.1128/mbio.00177-24 (PMC10936188; doi:10.1128/mbio.00177-24)
Supplement: Supplemental material — Supplemental tables and figures. [file mbio.00177-24-s0001.docx]

***Supplementary information for:***

**Active microbial population dynamics and life strategies drive the enhanced carbon use efficiency in high organic matter soils**

Qicheng Xu ^a, b, 1^, Ling Li ^a, 1^, Junjie Guo ^a^, Hanyue Guo ^a^, Manqiang Liu ^a, b^, Shiwei Guo ^a^, Yakov Kuzyakov ^c, d^, Ning Ling ^a, b, *^, Qirong Shen ^a, **^

^a^ *Jiangsu Collaborative Innovation Center for Solid Organic Waste Resource Utilization, Nanjing Agricultural University, Nanjing, 210095, China*

^b^ *Centre for Grassland Microbiome, State Key Laboratory of Grassland Agro-ecosystems, College of Pastoral Agricultural Science and Technology, Lanzhou University, Lanzhou, 730020, Gansu, China*

^c^ *Department of Soil Science of Temperate Ecosystems, Department of Agricultural Soil Science, University of Gottingen, 37077 Göttingen, Germany*

^d^ *Peoples Friendship University of Russia (RUDN University), 117198 Moscow, Russia*

1, Qicheng Xu and Ling Li contributed equally to this paper.

**Corresponding author:**

*Ning Ling

*E-mail address*: nling@njau.edu.cn; Tel. (+86) 25 84396853.

******Qirong Shen

*E-mail address*: shenqirong@njau.edu.cn; Tel. (+86) 25 84396291.

Table S1 The relative abundance (%) of Carbohydrate-Active enZYme (CAZYme) gene in the active communities based on the metagenomic sequencing.

|  | M | NPK |
| --- | --- | --- |
| GT | 42.44 ± 1.38 a | 40.62 ± 0.38 b |
| GH | 24.32 ± 0.57 b | 25.97 ± 0.42 a |
| CE | 13.94 ± 0.73 b | 16.53 ± 0.48 a |
| CBM | 14.31 ± 0.53 a | 12.03 ± 0.76 b |
| AA | 2.93 ± 0.59 a | 3.68 ± 0.22 a |
| PL | 1.81 ± 0.08 a | 0.94 ± 0.04 b |
| SLH | 0.14 ± 0.01 a | 0.13 ± 0.03 a |
| Dockerin | 0.09 ± 0.01 a | 0.08 ± 0.01 a |
| Cohesin | 0.02 ± 0.00 a | 0.02 ± 0.01 a |

Means (n = 3) ± standard deviations within a row followed by different letters indicate significance (*P* ≤ 0.05) using Student's t test.

Note: GH, glycoside hydrolase; GT, glycosyl transferase; PL, polysaccharide lyase; CE, carbohydrate esterase; AA, auxiliary activities; CBM, carbohydrate-binding module; SLH, S-layer homology domain; Dockerin, dockerin domain; Cohesin, cohesin domain.

Table S2 The information of different families of Carbohydrate-Active enZYmes (CAZYmes) analyzed with significance in this study.

| **Cazy family** | **Enzyme class** | **Process description** | **ECs in Family** | **Note** |
| --- | --- | --- | --- | --- |
| GH109 | Glycoside Hydrolases | NA | α-N-acetylgalactosaminidase (EC 3.2.1.49); β-N-acetylhexosaminidase (EC 3.2.1.52). | Created following Liu et al. (2007) Nature Biotechnol 25:454-64 (PMID: 17401360); the enzymes in this family display an unusual mechanism involving NAD+. |
| GH13 | Glycoside Hydrolases | Act on substrates containing α-glucoside linkages. | α-amylase (EC 3.2.1.1); pullulanase (EC 3.2.1.41); cyclomaltodextrin glucanotransferase (EC 2.4.1.19); cyclomaltodextrinase (EC 3.2.1.54); trehalose-6-phosphate hydrolase (EC 3.2.1.93); oligo-α-glucosidase (EC 3.2.1.10); maltogenic amylase (EC 3.2.1.133); neopullulanase (EC 3.2.1.135); α-glucosidase (EC 3.2.1.20); maltotetraose-forming α-amylase (EC 3.2.1.60); isoamylase (EC 3.2.1.68); glucodextranase (EC 3.2.1.70); maltohexaose-forming α-amylase (EC 3.2.1.98); maltotriose-forming α-amylase (EC 3.2.1.116); branching enzyme (EC 2.4.1.18); trehalose synthase (EC 5.4.99.16); 4-α-glucanotransferase (EC 2.4.1.25); maltopentaose-forming α-amylase (EC 3.2.1.-) ; amylosucrase (EC 2.4.1.4) ; sucrose phosphorylase (EC 2.4.1.7); malto-oligosyltrehalose trehalohydrolase (EC 3.2.1.141); isomaltulose synthase (EC 5.4.99.11); malto-oligosyltrehalose synthase (EC 5.4.99.15); amylo-α-1,6-glucosidase (EC 3.2.1.33); α-1,4-glucan: phosphate α-maltosyltransferase (EC 2.4.99.16); amino acid transporter; [retaining] sucrose 6(F)-phosphate phosphorylase (EC 2.4.1.329); [retaining] glucosylglycerol phosphorylase (EC 2.4.1.359); Glucosylglycerate phosphorylase (EC 2.4.1.352); [retaining] sucrose α-glucosidase (EC 3.2.1.48); oligosaccharide α-4-glucosyltransferase (EC 2.4.1.161); [retaining] α-amylase (EC 3.2.1.1). | New: many members have been assigned to subfamilies as described by Stam et al. (2006) Protein Eng Des Sel. 19, 555-562 (PMID: 17085431). |
| GT28 | Glycosyl Transferases | Galactolipid biosynthesis. | 1,2-diacylglycerol 3-ß-galactosyltransferase (EC 2.4.1.46); 1,2-diacylglycerol 3-ß-glucosyltransferase (EC 2.4.1.157);Ac-pentapeptide ß-N-acetylglucosaminyltransferase (EC 2.4.1.227); digalactosyldiacylglycerol synthase (EC 2.4.1.241). | Distantly related to family GT1. |
| GT9 | Glycosyl Transferases | Lipopolysaccharide synthesis. | Lipopolysaccharide N-acetylglucosaminyltransferase (EC 2.4.1.56); heptosyltransferase (EC 2.4.-.-). | NA |
| CBM44 | Carbohydrate-Binding Modules | NA | The C-terminal CBM44 module of the Clostridium thermocellum enzyme has been demonstrated to bind equally well cellulose and xyloglucan. | Created after Najmudin et al. J. Biol. Chem. (2006) 281:8815-8828 (PMID: 16314409). |
| GH74 | Glycoside Hydrolases | Glucan degradation. | Endoglucanase (EC 3.2.1.4); oligoxyloglucan reducing end-specific cellobiohydrolase (EC 3.2.1.150); xyloglucanase (EC 3.2.1.151). | NA |
| GH1 | Glycoside Hydrolases | Cleave various sugars. | ß-glucosidase (EC 3.2.1.21); ß-galactosidase (EC 3.2.1.23); ß-mannosidase (EC3.2.1.25); ß-glucuronidase (EC 3.2.1.31); ß-xylosidase (EC 3.2.1.37); ß-D-fucosidase (EC 3.2.1.38); phlorizin hydrolase (EC 3.2.1.62); exo-ß-1,4-glucanase (EC 3.2.1.74); 6-phospho-ß-galactosidase (EC 3.2.1.85); 6-phospho-ß-glucosidase (EC 3.2.1.86); strictosidine ß-glucosidase (EC3.2.1.105); lactase (EC 3.2.1.108); amygdalin ß-glucosidase (EC 3.2.1.117); prunasin ß-glucosidase (EC 3.2.1.118); vicianin hydrolase (EC3.2.1.119); raucaffricine ß-glucosidase (EC 3.2.1.125); thioglucosidase (EC 3.2.1.147); ß-primeverosidase (EC 3.2.1.149); isoflavonoid 7-O-ß-apiosyl-ß-glucosidase (EC 3.2.1.161); ABA-specific ß-glucosidase (EC 3.2.1.175); DIMBOA ß-glucosidase (EC 3.2.1.182); ß-glycosidase (EC 3.2.1.-); hydroxyisourate hydrolase (EC 3.-.-.-); ß-rutinosidase /a-L-rhamnose-(1,6)-ß-D-glucosidase (EC 3.2.1.-). | NA |
| CBM50 | Carbohydrate-Binding Modules | NA | Modules of approx. 50 residues found attached to various enzymes from families GH18, GH19, GH23, GH24, GH25 and GH73, i.e. enzymes cleaving either chitin or peptidoglycan. Binding to chitopentaose demonstrated in the case of Pteris ryukyuensis chitinase A [Ohnuma T et al. (2008) J. Biol. Chem. 283:5178-87 (PMID: 18083709)]. CBM50 modules are also found in a multitude of other enzymes targeting the petidoglycan such as peptidases and amidases. These enzymes are not reported in the list below. | Also known as LysM domains. |
| GH23 | Glycoside Hydrolases | Peptidoglycan degradation, chitin degradation. | Lysozyme type G (EC 3.2.1.17); peptidoglycan lyase (EC 4.2.2.;n1) also known in the literature as peptidoglycan lytic transglycosylase; chitinase (EC 3.2.1.14). | Corresponds to family 1 of the peptidoglycan lytic transglycosylases described by N.T. Blackburn and A.J. Clarke (2001) J. Mol. Evol. 52, 78-84; Note that peptidoglycan lytic transglycosylases cleave peptidoglycan without intervention of a water molecule. |
| GH43 | Glycoside Hydrolases | NA | β-xylosidase (EC 3.2.1.37); α-L-arabinofuranosidase (EC 3.2.1.55); xylanase (EC 3.2.1.8); α-1,2-L-arabinofuranosidase (EC 3.2.1.-); exo-α-1,5-L-arabinofuranosidase (EC 3.2.1.-); [inverting] exo-α-1,5-L-arabinanase (EC 3.2.1.-); β-1,3-xylosidase (EC 3.2.1.-); [inverting] exo-α-1,5-L-arabinanase (EC 3.2.1.-); [inverting] endo-α-1,5-L-arabinanase (EC 3.2.1.99); exo-β-1,3-galactanase (EC 3.2.1.145); β-D-galactofuranosidase (EC 3.2.1.146). | Many members have been assigned to subfamilies as described by Mewis et al. (2016) Appl. Environm. Microbiol. 82:1686-1692 (PMID: 26729713). |
| GT4 | Glycosyl Transferases | NA | Sucrose synthase (EC 2.4.1.13); sucrose-phosphate synthase (EC 2.4.1.14); α-glucosyltransferase (EC 2.4.1.52); lipopolysaccharide N-acetylglucosaminyltransferase (EC 2.4.1.56); phosphatidylinositol α-mannosyltransferase (EC 2.4.1.57); GDP-Man: Man1GlcNAc2-PP-dolichol α-1,3-mannosyltransferase (EC 2.4.1.132); GDP-Man: Man3GlcNAc2-PP-dolichol/Man4GlcNAc2-PP-dolichol α-1,2-mannosyltransferase (EC 2.4.1.131); digalactosyldiacylglycerol synthase (EC 2.4.1.141); 1,2-diacylglycerol 3-glucosyltransferase (EC 2.4.1.157); diglucosyl diacylglycerol synthase (EC 2.4.1.208); trehalose phosphorylase (EC 2.4.1.231); NDP-Glc: α-glucose α-glucosyltransferase / α,α-trehalose synthase (EC 2.4.1.245); GDP-Man: Man2GlcNAc2-PP-dolichol α-1,6-mannosyltransferase (EC 2.4.1.257); UDP-GlcNAc: 2-deoxystreptamine α-N-acetylglucosaminyltransferase (EC 2.4.1.283); UDP-GlcNAc: ribostamycin α-N-acetylglucosaminyltransferase (EC 2.4.1.285); UDP-Gal α-galactosyltransferase (EC 2.4.1.-); UDP-Xyl α-xylosyltransferase (EC 2.4.2.-); UDP-GlcA α-glucuronyltransferase (EC 2.4.1.-); UDP-Glc α-glucosyltransferase (EC 2.4.1.-); UDP-GalNAc: GalNAc-PP-Und α-1,3-N-acetylgalactosaminyltransferase (EC 2.4.1.306); UDP-GalNAc: N,N'-diacetylbacillosaminyl-PP-Und α-1,3-N-acetylgalactosaminyltransferase (EC 2.4.1.290); ADP-dependent α-maltose-1-phosphate synthase (2.4.1.342); [retaining] UDP-GlcNAc: polypeptide α-N-acetylglucosaminyltransferase (EC 2.4.1.-); UDP-GlcNAc: α-N-acetylglucosaminyltransferase (EC 2.4.1.-). | NA |
| CBM32 | Carbohydrate-Binding Modules | NA | Binding to galactose and lactose has been demonstrated for the module of Micromonospora viridifaciens sialidase (PMID: 16239725). Binding to polygalacturonic acid has been shown for a Yersinia member (PMID: 17292916). Binding to LacNAc (β-D-galactosyl-1,4-β-D-N-acetylglucosamine) has been shown for an N-acetylglucosaminidase from Clostridium perfingens (PMID: 16990278). | Formerly known as X56 modules. Distantly related to CBM6 modules and to Anguilla anguilla agglutinin. |
| GT2 | Glycosyl Transferases | NA | Cellulose synthase (EC 2.4.1.12); chitin synthase (EC 2.4.1.16); dolichyl-phosphate β-D-mannosyltransferase (EC 2.4.1.83); dolichyl-phosphate β-glucosyltransferase (EC 2.4.1.117); N-acetylglucosaminyltransferase (EC 2.4.1.-); N-acetylgalactosaminyltransferase (EC 2.4.1.-); hyaluronan synthase (EC 2.4.1.212); chitin oligosaccharide synthase (EC 2.4.1.-); β-1,3-glucan synthase (EC 2.4.1.34); β-1,4-mannan synthase (EC 2.4.1.-); β-mannosylphosphodecaprenol-mannooligosaccharide α-1,6-mannosyltransferase (EC 2.4.1.199); UDP-Galf: rhamnopyranosyl-N-acetylglucosaminyl-PP-decaprenol β-1,4/1,5-galactofuranosyltransferase (EC 2.4.1.287); UDP-Galf: galactofuranosyl-galactofuranosyl-rhamnosyl-N-acetylglucosaminyl-PP-decaprenol β-1,5/1,6-galactofuranosyltransferase (EC 2.4.1.288); dTDP-L-Rha: N-acetylglucosaminyl-PP-decaprenol α-1,3-L-rhamnosyltransferase (EC 2.4.1.289); alternating β-1,3/4-N-acetylmannan synthase (2.4.1.-); UDP-GlcA: N-acetylglucosaminyl-proteoglycan β-1,4-glucuronosyltransferase (EC 2.4.1.225). | Distant similarity to families GT12, GT21, GT27, GT55, GT81 etc. |
| GH3 | Glycoside Hydrolases | Glu for hydrolases (experimental); histidine for phosphorylases (experimental). | β-glucosidase (EC [3.2.1.21](http://www.enzyme-database.org/query.php?ec=3.2.1.21)); xylan 1,4-β-xylosidase (EC [3.2.1.37](http://www.enzyme-database.org/query.php?ec=3.2.1.37)); β-glucosylceramidase (EC [3.2.1.45](http://www.enzyme-database.org/query.php?ec=3.2.1.45)); β-N-acetylhexosaminidase (EC [3.2.1.52](http://www.enzyme-database.org/query.php?ec=3.2.1.52)); α-L-arabinofuranosidase (EC [3.2.1.55](http://www.enzyme-database.org/query.php?ec=3.2.1.55)); glucan 1,4-β-glucosidase (EC [3.2.1.74](http://www.enzyme-database.org/query.php?ec=3.2.1.74)); isoprimeverose-producing oligoxyloglucan hydrolase (EC [3.2.1.120](http://www.enzyme-database.org/query.php?ec=3.2.1.120)); coniferin β-glucosidase (EC [3.2.1.126](http://www.enzyme-database.org/query.php?ec=3.2.1.126)); exo-1,3-1,4-glucanase (EC [3.2.1.-](http://www.enzyme-database.org/query.php?ec=3.2.1.*)); β-N-acetylglucosaminide phosphorylases (EC [2.4.1.-](http://www.enzyme-database.org/query.php?ec=2.4.1.*)); β-1,2-glucosidase (EC [3.2.1.-](http://www.enzyme-database.org/query.php?ec=3.2.1.*)); β-1,3-glucosidase (EC [3.2.1.-](http://www.enzyme-database.org/query.php?ec=3.2.1.*)); xyloglucan-specific exo-β-1,4-glucanase / exo-xyloglucanase (EC [3.2.1.155](http://www.enzyme-database.org/query.php?ec=3.2.1.155)); stevioside-β-1,2-glucosidase (EC [3.2.1.-](http://www.enzyme-database.org/query.php?ec=3.2.1.*)); lichenase / endo-β-1,3-1,4-glucanase (EC [3.2.1.73](http://www.enzyme-database.org/query.php?ec=3.2.1.73)); protodioscin 26-O-β-D-glucosidase (EC [3.2.1.186](http://www.enzyme-database.org/query.php?ec=3.2.1.186)); β-glucuronidase (EC [3.2.1.31](http://www.enzyme-database.org/query.php?ec=3.2.1.31)). | A recent paper by Macdonald et al. (PMID=25533455) has shown that some enzymes that cleave b-hexosaminides are in fact retaining b-glycoside phosphorylases. |
| CE1 | Carbohydrate Esterases | Utilize the canonical serine hydrolase mechanism. | Acetyl xylan esterase (EC 3.1.1.72); cinnamoyl esterase (EC 3.1.1.-); feruloyl esterase (EC 3.1.1.73); carboxylesterase (EC 3.1.1.1); S-formylglutathione hydrolase (EC 3.1.2.12); diacylglycerol O-acyltransferase (EC 2.3.1.20); trehalose 6-O-mycolyltransferase (EC 2.3.1.122). | Family CE1 also contains lots of other esterases such as PHB depolymerases. |
| CE7 | Carbohydrate Esterases | NA | Acetyl xylan esterase (EC 3.1.1.72); cephalosporin-C deacetylase (EC 3.1.1.41). | NA |
| GH102 | Glycoside Hydrolases | Peptidoglycan degradation. | Peptidoglycan lytic transglycosylase (EC 3.2.1.-). | Corresponds to family 2 of the peptidoglycan lytic transglycosylases described by N.T. Blackburn and A.J. Clarke (2001) J. Mol. Evol. 52, 78-84. |
|  | | | | |

Table S3 The relative abundance (%) of Carbohydrate-Active enZYme (CAZYme) gene in *Sphingomonas* genus based on the metagenomic sequencing.

|  | M | NPK |
| --- | --- | --- |
| GT | 44.13±1.78 a | 34.87±1.48 b |
| GH | 26.83±1.82 b | 32.08±1.17 a |
| CE | 18.33±0.62 b | 24.49±1.32 a |
| PL | 3.96±1.34 a | 3.20±1.18 a |
| AA | 3.26±1.24 a | 2.82±0.46 a |
| CBM | 3.49±0.65 a | 2.55±0.67 a |

Means (n = 3) ± standard deviations within a row followed by different letters indicate significance (*P* ≤ 0.05) using Student's t test.

Note: GH, glycoside hydrolase; GT, glycosyl transferase; PL, polysaccharide lyase; CE, carbohydrate esterase; AA, auxiliary activities; CBM, carbohydrate-binding module; SLH, S-layer homology domain; Dockerin, dockerin domain; Cohesin, cohesin domain.

Table S4 Chemical properties of different fertilized soils.

| Soil attributes | Treatment | | | |
| --- | --- | --- | --- | --- |
|  | Control | NPK | M | NPKM |
| pH | 4.80±0.08c | 4.40±0.09d | 6.27±0.06a | 5.62±0.02b |
| SOC (g kg^-1^) | 7.40±0.13c | 9.20±0.80b | 11.73±0.38a | 11.70±0.32a |
| TN (g kg^-1^) | 0.92±0.02c | 1.07±0.08b | 1.38±0.02a | 1.36±0.05a |
| TP (g kg^-1^) | 0.56±0.03d | 0.77±0.05c | 1.51±0.15b | 2.10±0.10a |

Means (*n* = 3) ± standard deviations followed by dissimilar letters indicate significance (*P* ≤ 0.05) across the treatments.

Table S5 Soil biological properties for the calculations of conversion factors.

| Parameters | Control | NPK | M | NPKM |
| --- | --- | --- | --- | --- |
| MBC (ug g^-1^) | 112.60 ± 11.18 | 127.35 ± 6.23 | 151.52 ± 15.38 | 140.58 ± 6.46 |
| DNA (ug g^-1^) | 1.27 ± 0.08 | 1.35 ± 0.22 | 5.63 ± 0.79 | 3.8 ± 0.33 |
| f_DNA_ | 88.22 ± 8.76 | 94.17 ± 4.61 | 26.91 ± 2.73 | 36.86 ± 1.69 |


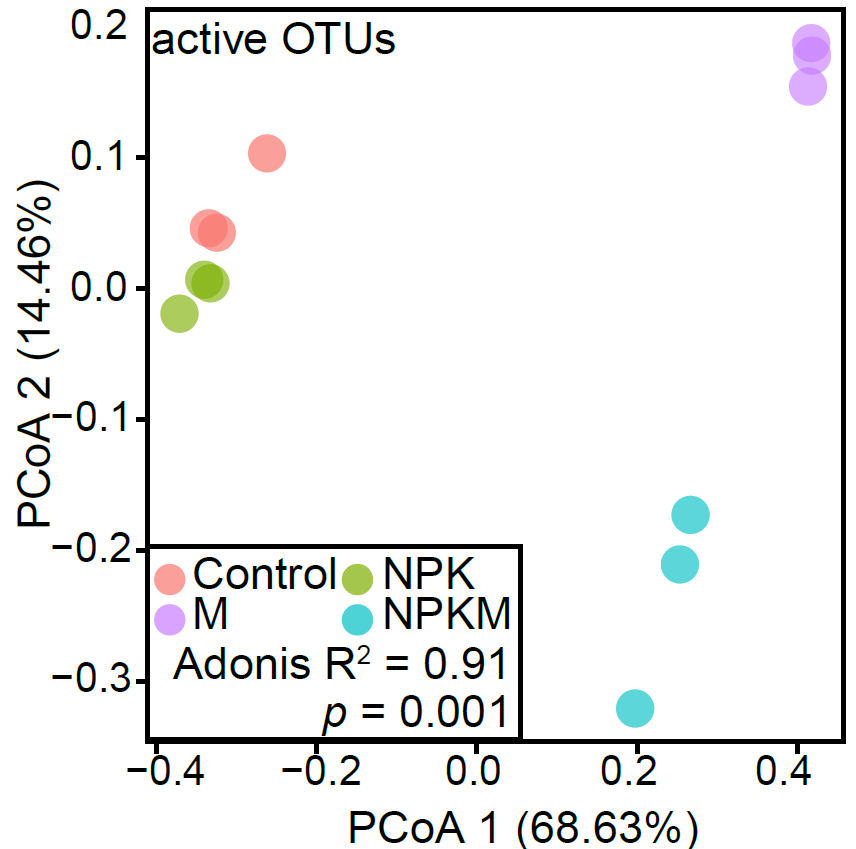


Fig. S1 Unconstrained principal coordinates analysis (PCoA) with Bray-Curtis distances are shown based on the EAF-values of active OTUs.


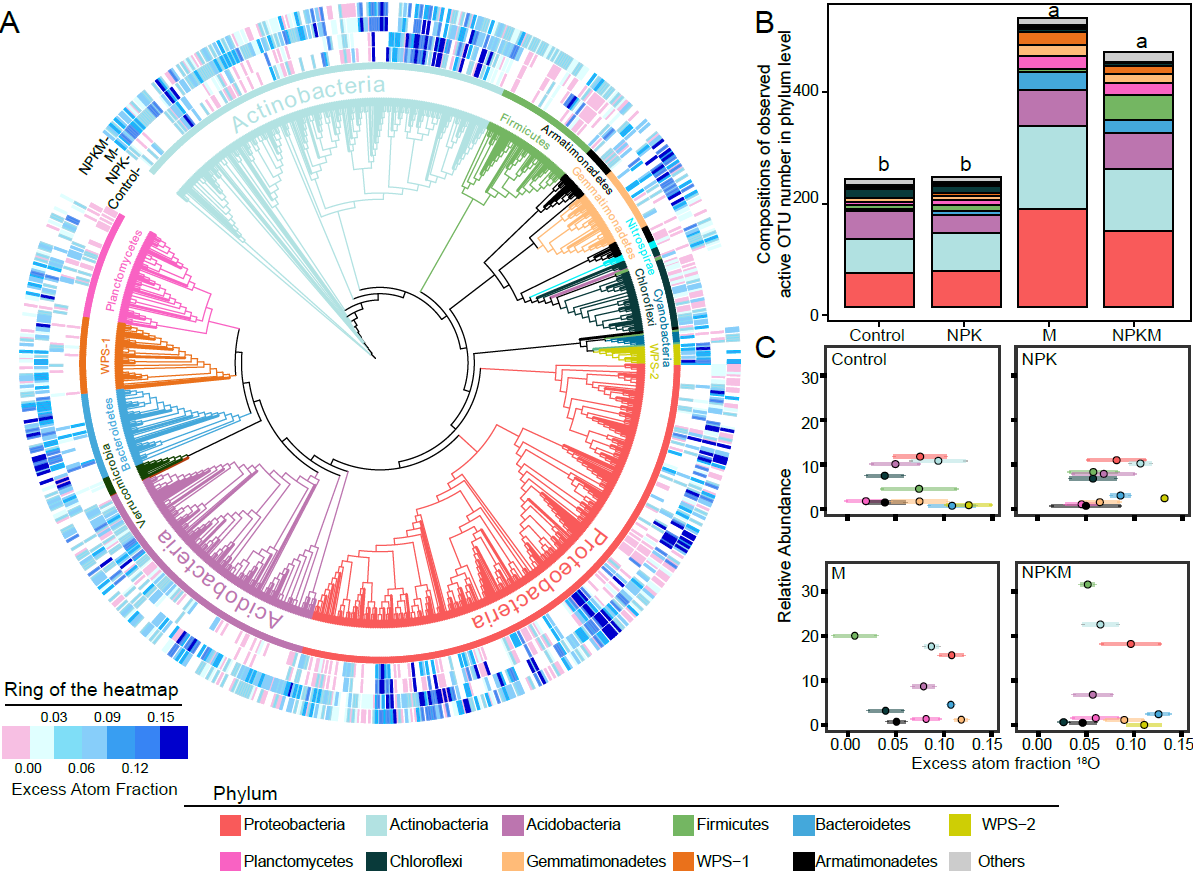


Fig. S2 Phylogenetic tree and heat map of ^18^O incorporation by bacterial taxa (A). The ring of the heatmap corresponds to the excess atom fraction of the ^18^O (EAF-^18^O) values of labeled taxa in soils under the Control and NPK-, M- and NPKM-fertilization. The absolute compositions of observed active OTUs at the phylum level (phylum assignment provided) are indicated (B). The relationships between the original relative abundance of each phylum and the mean excess atom fraction of ^18^O (EAF-^18^O) values are displayed for the Control and NPK-, M- and NPKM-fertilization, respectively (C). The ^18^O labeling degree of a given species was independent of its original relative abundance in the soil microbial reservoir. Error bars: 95% confidence intervals. Control: no fertilization over 30 years; NPK: addition of mineral fertilizers only; M: manure addition; NPKM: addition of both mineral fertilizers and manure.


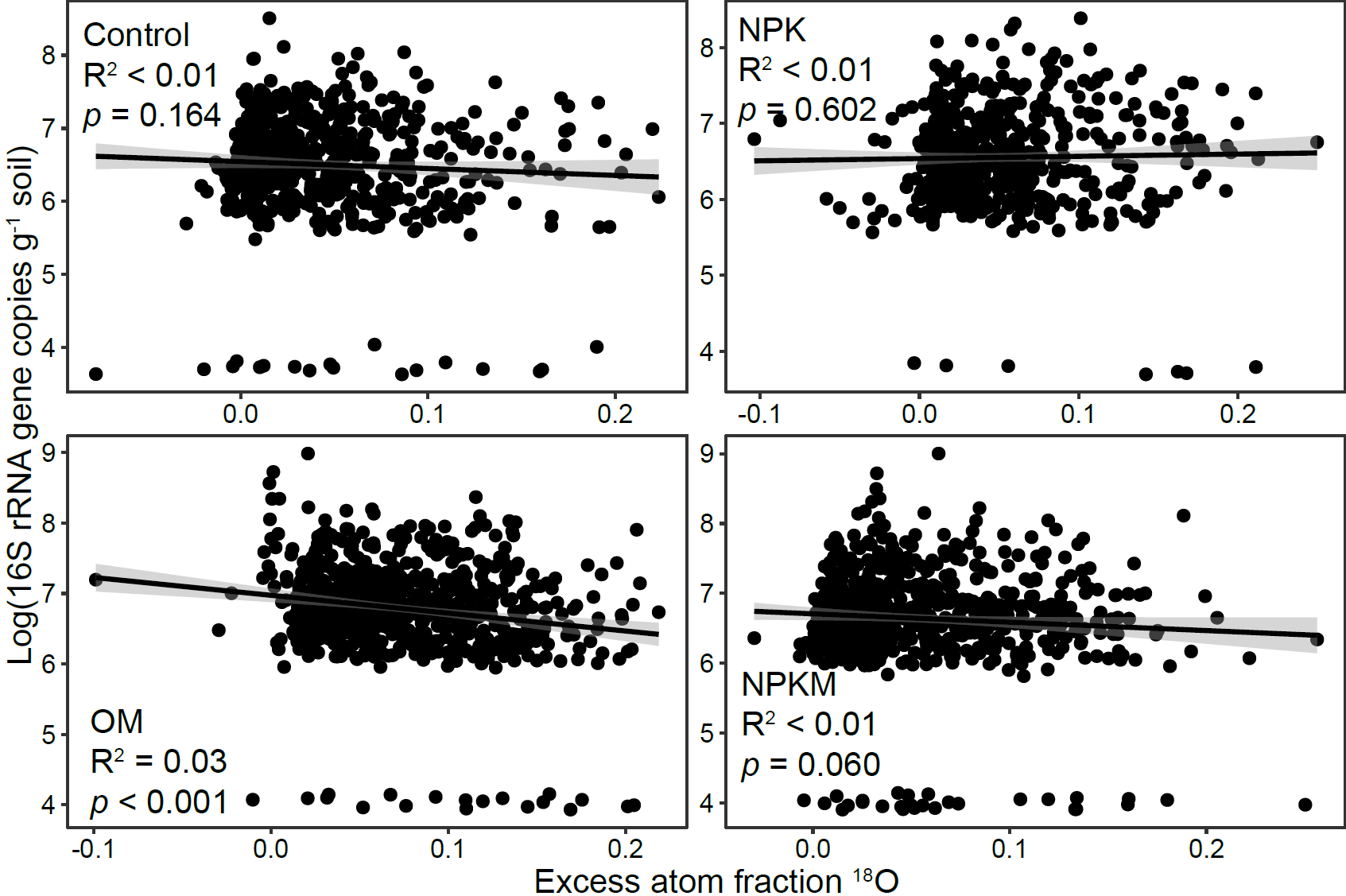


Fig. S3 The relationships between the original absolute abundance of each taxon and its excess atom fraction of ^18^O (EAF-^18^O) values.


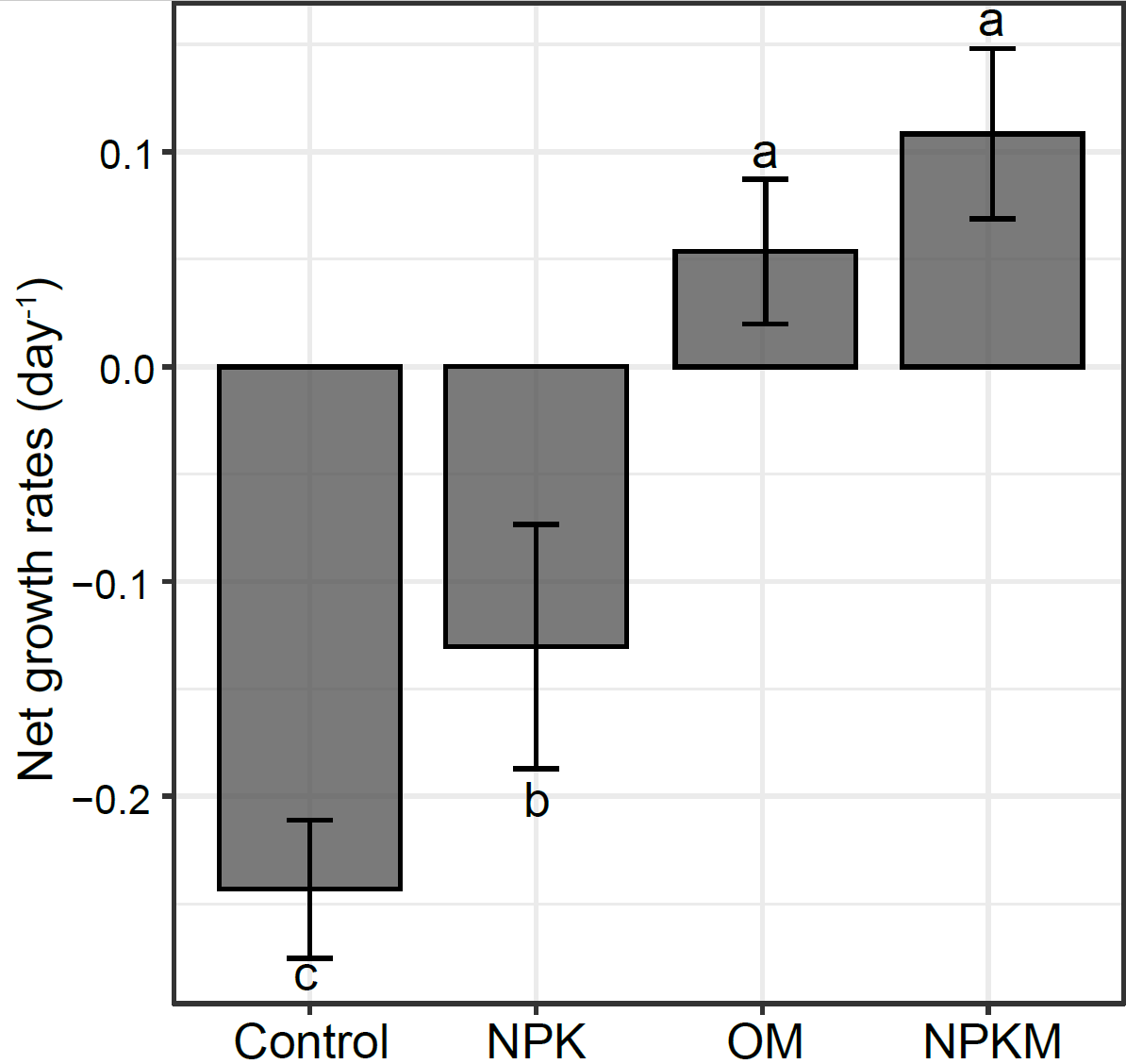


Fig. S4 The net growth rates of *Sphingomonas* inferred by qSIP.


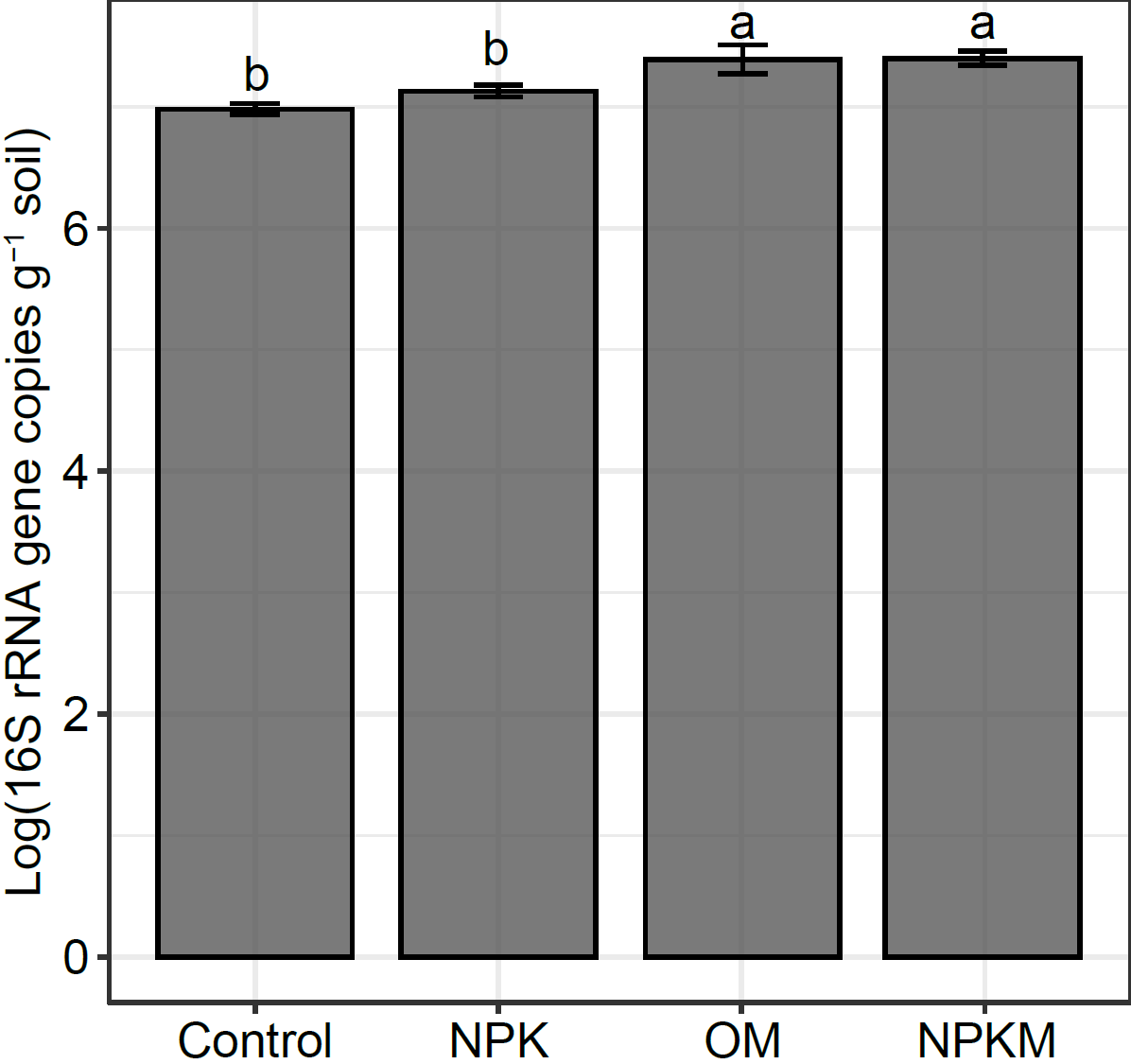


Fig. S5 The absolute abundance of *Sphingomonas*.


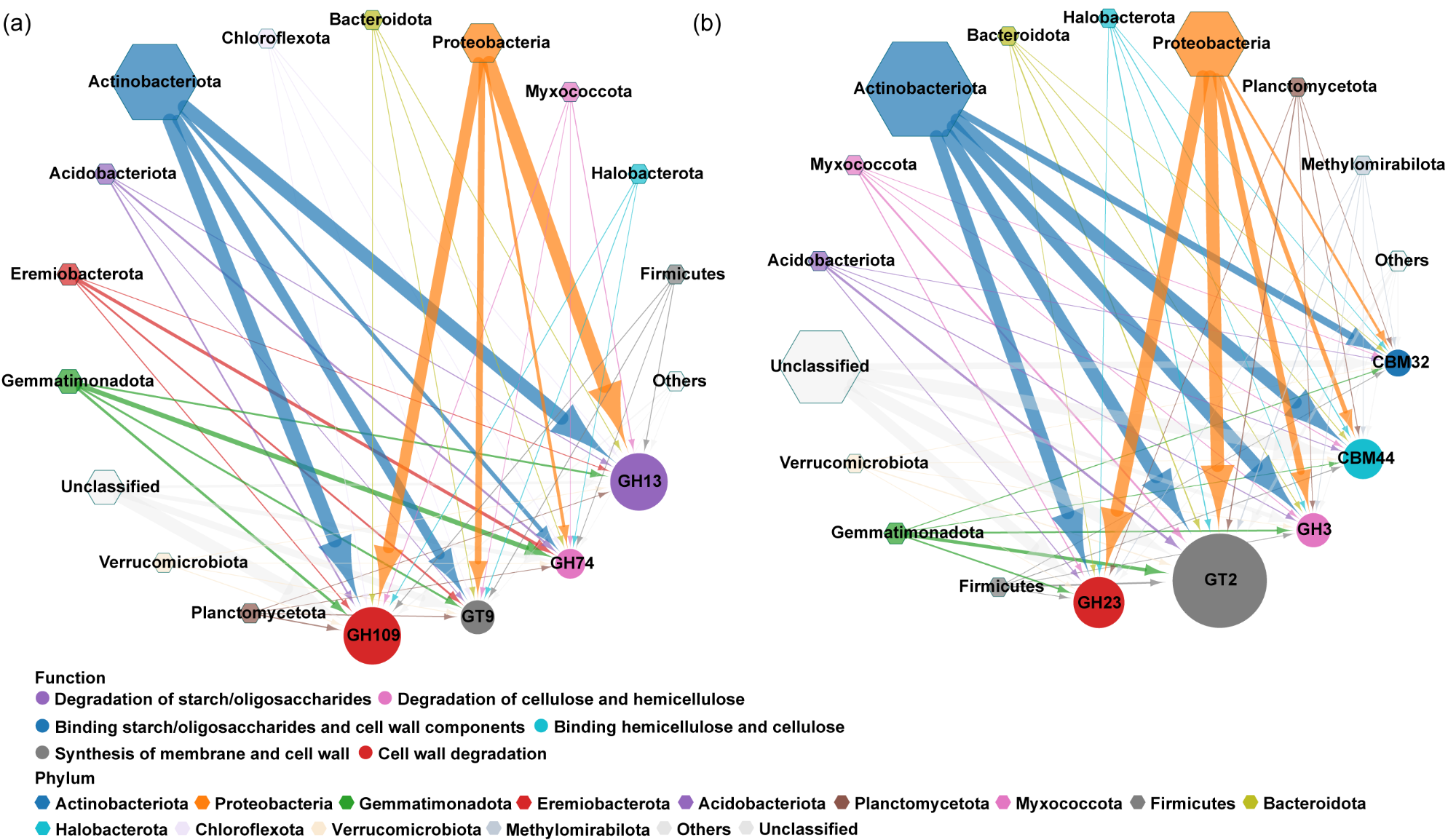


Fig. S6 Taxa driving enriched carbon utilization related genes in (a) NPK and (b) M. Enzyme substrates are provided. The width of each ribbon represents the average abundance of the genes driven by a given phylum.


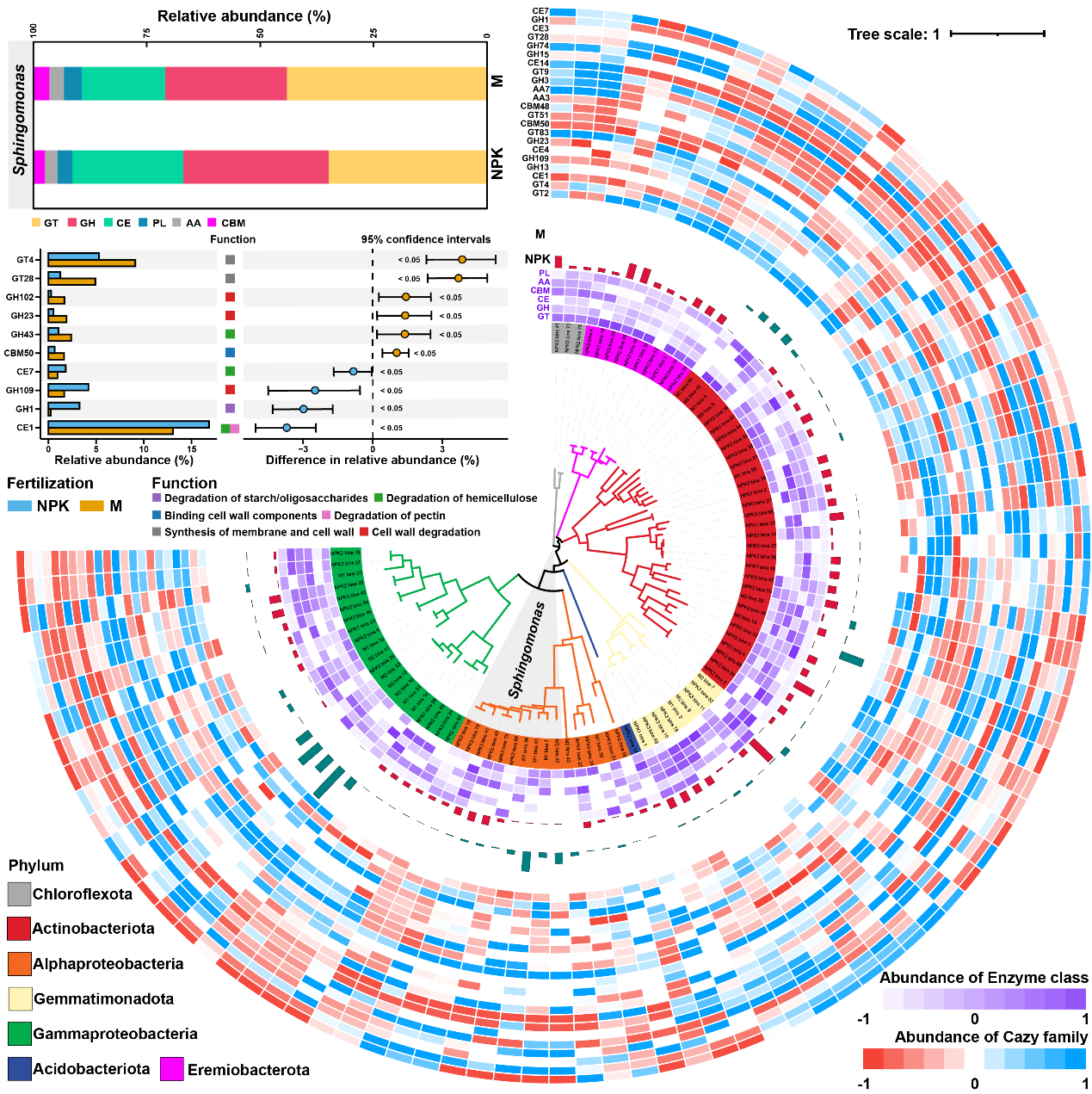


Fig. S7 Carbon utilization related genes at genome level. Phylogenetic tree of metagenome assembled genomes (MAG), taxonomic information of MAGs, abundances of carbon utilization related genes at the enzyme class level, relative abundances of MAGs and the abundances of carbon utilization related genes at the enzyme family level are shown from the inner to outer circles. Relative abundances of carbon utilization related genes of *Sphingomonas* at the enzyme class level is focused on, and genes with significant differences in abundances at the enzyme family level as well as the related substrates are provided (upper left of the figure).

Note: In total, 91 metagenome assembled genomes (MAGs, i.e., medium- and high-quality bins with completeness >70% and contamination <10%) were recovered from the metagenomic data. The cumulative abundance of these MAGs accounted for more than 40% of the total microbial abundance. These MAGs belonged to Proteobacteria (37 of 91), Actinobacteria (31 of 91), Eremiobacterota (WPS-2, 10 of 91), Gemmatimonadetes (9 of 91), Chloroflexi (3 of 91) and Acidobacteria (1 of 91).


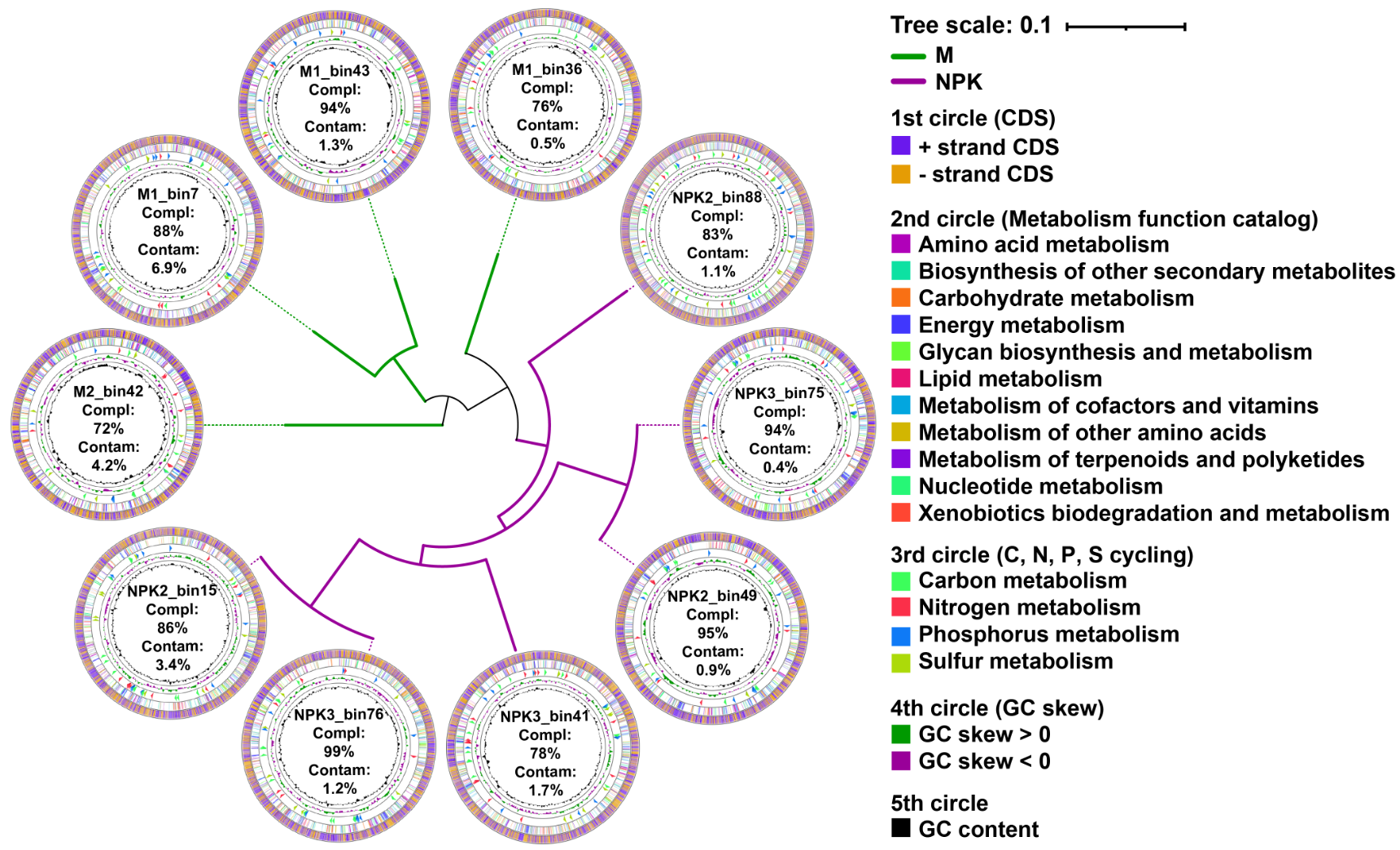


Fig. S8 Genome map of *Sphingomonas* combined with information on metabolism function catalog, nutrient cycling and GC content.


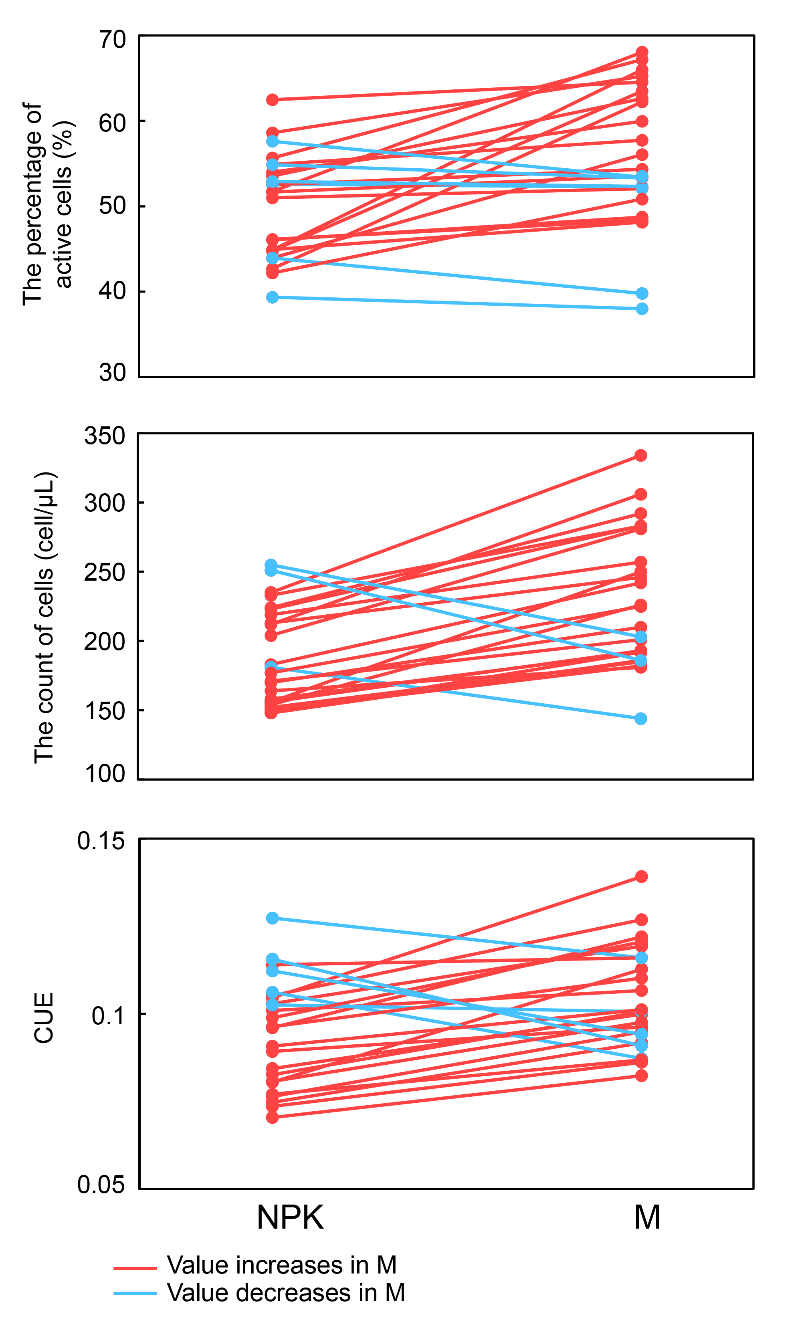


Fig. S9 The active cells, cell count and CUE of 24 *Sphingomonas* isolates when growing in the sterilized soil supernatant of M soils and NPK soils.

**Notes:**

The paired sample t-test was used to test the parameters. 24 *Sphingomonas* isolates were different species, implying that each isolate's response under M and NPK conditions might be different, making it essential to consider the paired nature of the data. The results showed that all parameters were significantly (*P* < 0.05) higher in M condition.

The CUE was calculated based on the cell counts and respiration according to the previous study (Stone et al.,2021). The genome size of *Sphingomonas* was predicted by the GenBank database. The cytoplasmic mass of *Sphingomonas* was estimated utilizing the allometric scaling relationship between genome size and cytoplasmic mass (West and Brown, 2005). Based on the carbon content per unit biomass of microbes, the growth of microbes per unit time was estimated. Combining the respiratory carbon loss of microbes per unit time, the CUE of *Sphingomonas* was calculated as the following table.

Calculating the CUE of *Sphingomonas*

| Parameters | Formulas | Clarification |
| --- | --- | --- |
| Cell mass of species i (Mi, g) | $\text{log}_{\text{10}}\text{(Mi)=}\frac{\text{log10(Gi)-9.4}}{\text{0.24}}\text{ }\text{①}$ | Gi refers to the genome length (bp) of species i. |
| Amount of newly microbial biomass carbon by species i within time t (Pi，g C battle-1 t-1) | $\text{P}_{\text{i}}\text{=Cell number×}\text{M}_{\text{i}}\text{×0.2 }\text{②}$ | 0.2 indicates that about 20% of the cell mass is derived from carbon 0.2 (Bratbak and Dundas, 1984). |
| CUE for species i | $\text{CUE=}\frac{\text{Pi}}{\text{Pi+R}}\text{ }\text{③}$ | R is the total amount of CO2 respired by microorganisms with time t (g C battle-1). |


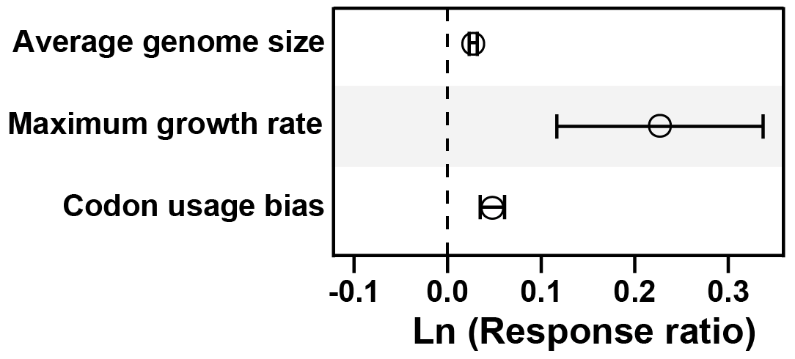


Fig. S10 The average genome size, maximum growth rate and codon usage bias of active microbes in organically fertilized soils compared with mineral fertilized soils. A response ratio > 0 indicates a higher value in organically fertilized soils.

Note: A t-test was used to compare the response ratios against a null hypothesis value of zero. All the response ratios were significantly (*P* < 0.05) different from zero.


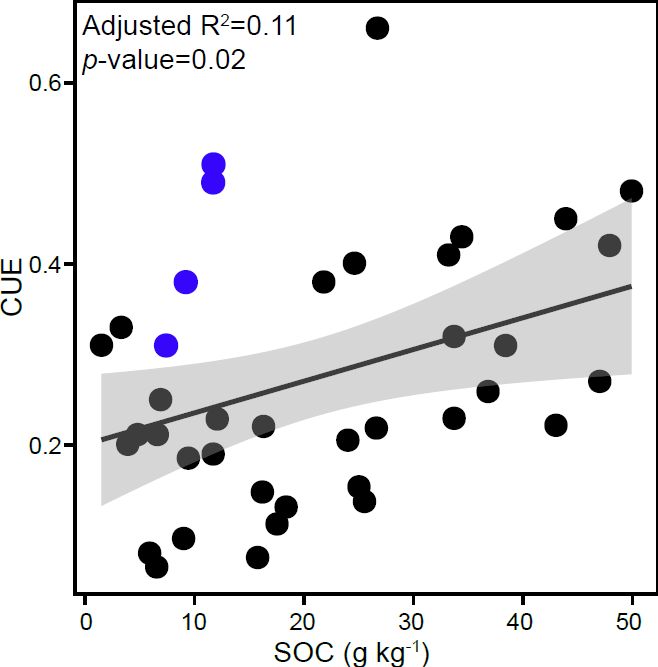


Fig. S11 Relationships between the SOC values and the microbial carbon use efficiencies (CUEs). The values come from this study (blue circles) and from the literature (Spohn et al., 2016a; Spohn et al., 2016b; Zheng et al., 2019; Canarini et al., 2020; Zhran et al., 2020, black circles). All studies calculated CUE based on H_2_^18^O.


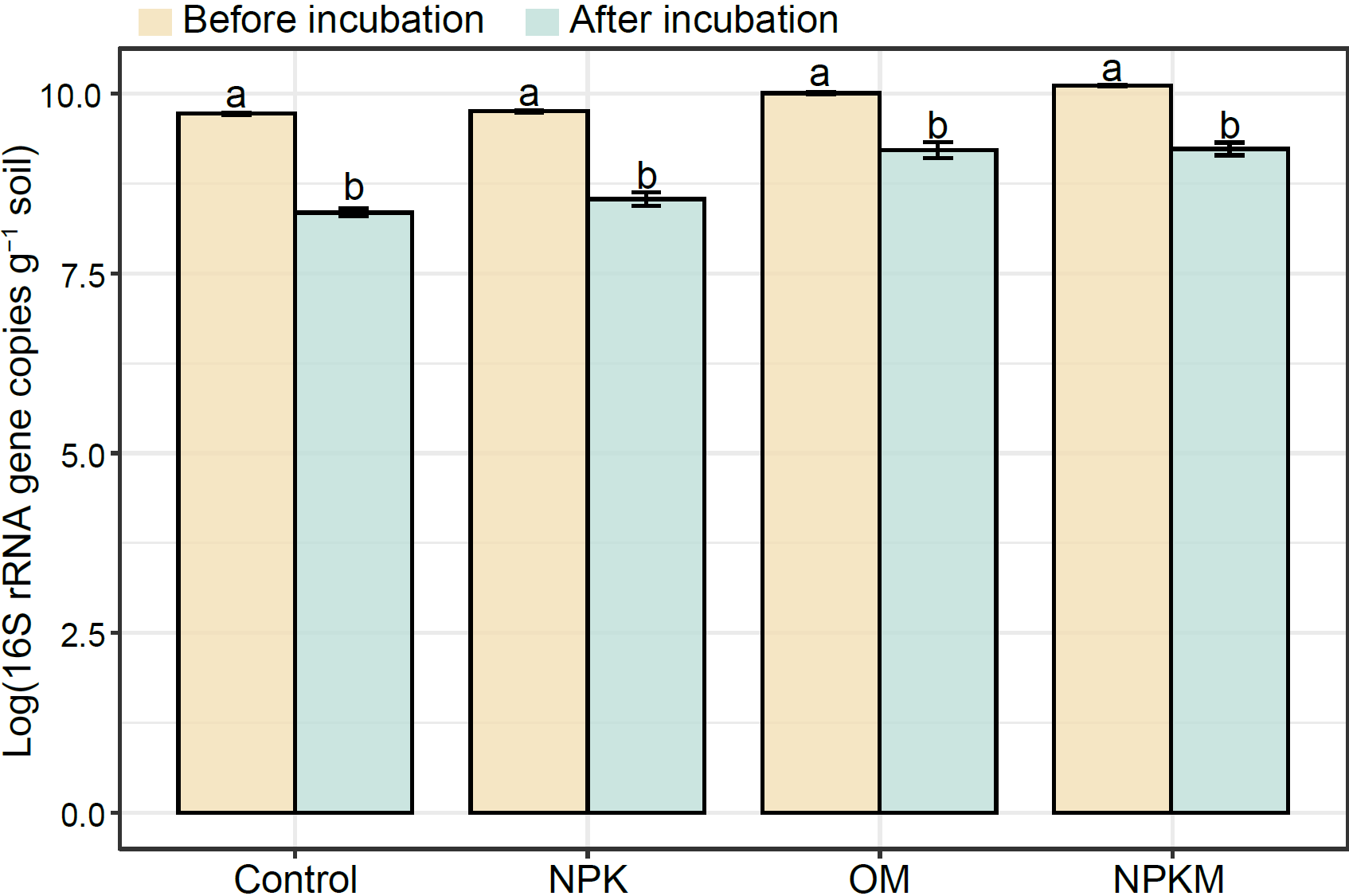


Fig. S12 The 16S rRNA gene copy number in soils before and after incubation.


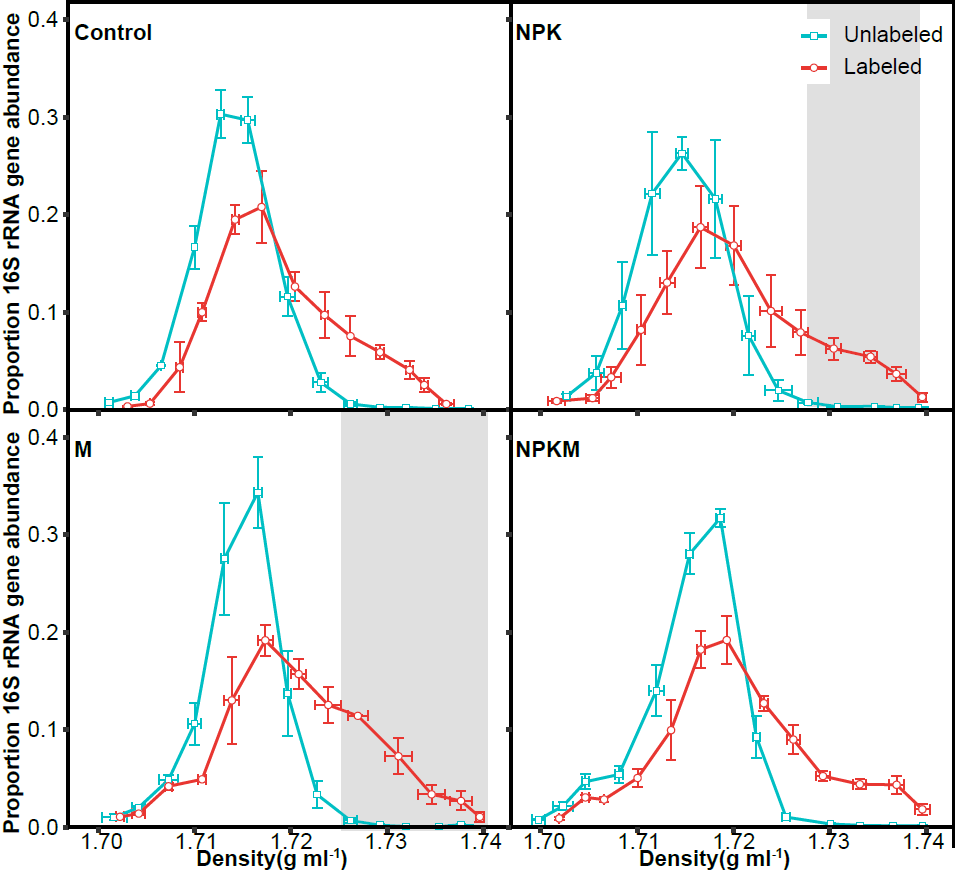


Fig. S13 Quantification of 16S rRNA gene copies across CsCl density gradient fractions. The ^18^O water replicates are represented by red solid lines with squares, the unlabeled replicates (controls) by cyan solid lines with circles. y axis: relative abundance of 16S rRNA genes quantified with qPCR. Control: no fertilization; NPK: addition of mineral fertilizers only; M: manure addition; NPKM: addition of both mineral fertilizers and manure. Error bars: standard deviations. All samples were used for qSIP sequencing; samples in grey area were used for metagenomics sequencing.
